# Global Gac/Rsm regulatory system activates the biosynthesis of mupirocin by controlling the MupR/I quorum sensing system in *Pseudomonas* sp. NCIMB 10586

**DOI:** 10.1128/aem.01896-24

**Published:** 2025-01-23

**Authors:** Yuyuan Cai, Peng Huang, Vittorio Venturi, Runyao Xiong, Zheng Wang, Wei Wang, Xianqing Huang, Hongbo Hu, Xuehong Zhang

**Affiliations:** 1 State Key Laboratory of Microbial Metabolism, School of Life Sciences and Biotechnology, Shanghai Jiao Tong University 200639 , Shanghai, China; 2 International Centre for Genetic Engineering and Biotechnology 18470 , Trieste, Italy; 3 African Genome Center, University Mohammed VI Polytechnic 479571 , Ben Guerir, Morocco; 4 National Experimental Teaching Center for Life Sciences and Biotechnology, Shanghai Jiao Tong University 12474 , Shanghai, China; Indiana University Bloomington, Bloomington, Indiana, USA

**Keywords:** GacS/GacA two-component system, mupirocin, quorum sensing, RsmY/Z, RsmA/E/I/F/N

## Abstract

The biosynthesis of mupirocin, a clinically significant antibiotic produced by *Pseudomonas* sp. NCIMB 10586, is activated by the *N*-acyl homoserine lactone (AHL) MupR/I quorum sensing (QS) system. However, to date, limited research has focused on the influence of global regulators such as the GacS/A two-component system (TCS) on the MupR/I QS system or mupirocin biosynthesis. In this study, we characterized the regulatory components of the Gac/Rsm transduction system in the mupirocin-producing model strain NCIMB 10586 and investigated their interconnection with the MupR/I QS circuit and subsequent mupirocin biosynthesis. The production of mupirocin was hampered by either *gacS* inactivation, *gacA* inactivation, or the double-mutant of the sRNAs ( RsmY and RsmZ). Similarly, the expressions of *mupR* and *mupI*, and AHL synthesis significantly decreased in *gacS, gacA,* or *rsmY/Z* mutants, indicating that the GacS/A system stimulates mupirocin biosynthesis via the MupR/I QS system. Five CsrA family proteins, RsmA/E/I/F/N, were found in strain NCIMB 10586, and the single and multiple mutants of *rsmA/E/I/F/N* showed different phenotypes with respect to mupirocin production. Our results revealed that mupirocin biosynthesis was likely to be negatively regulated by RsmA/E/I, but positively regulated by RsmF. Additionally, the RsmF protein was shown to interact with the 5’ leader of *mupR* mRNA. In summary, the Gac/Rsm system positively regulates the biosynthesis of mupirocin mainly through the MupR/I QS system, and the model of the regulatory mechanism is proposed. The elucidation of the Gac/Rsm-MupR/I regulatory pathway could help devise ways for improving mupirocin production through genetic engineering.

**IMPORTANCE:**

The Gac/Rsm regulatory system plays a global regulatory role in bacterial physiology and metabolism, including secondary metabolism. Mupirocin is a clinically important antibiotic, produced by *Pseudomonas* sp. NCIMB 10586, whose biosynthesis is activated by the MupR/I quorum sensing system. Global regulators have important impacts on the gene expression of secondary metabolic gene clusters and QS genes, and the GacS/A two-component system is one of the main regulators across *Pseudomonas* species, which significantly influences antibiotic production. Our study presented that the expressions of QS genes and *mup* gene cluster were downregulated in *gacS, gacA,* or *rsmY/Z* mutants compared to the wild-type. The inactivation of *rsmA/E/I/F/N* in NCIMB 10586, encoding CsrA family proteins, showed different regulatory traits of mupirocin production, in which the RsmF protein could interact with the 5’ UTR region of *mupR* mRNA. These findings provide the understanding of the regulatory role of Gac/Rsm on mupirocin biosynthesis and *mupR/I* QS system and lay foundations for further improving mupirocin production.

## INTRODUCTION

The polyketide antibiotic mupirocin was discovered in *Pseudomonas* sp. NCIMB 10586, previously named as *Pseudomonas fluorescens* NCIMB 10586 (1). It is currently applied as a topical antimicrobial treatment for nasal infections (2) due to the characteristic of blocking protein synthesis by competitively targeting isoleucyl-tRNA synthase (3). Mupirocin is currently used as a treatment strategy in eliminating methicillin-resistant *Staphylococcus aureus* (MRSA), which is responsible for an increasing number of skin infections and is, therefore, a cause for concern (4). Recently, mupirocin has been extensively used in combination pharmacotherapy for the prevention of topical infections. El-Sayed et al. reported that the biosynthesis of mupirocin in the strain NCIMB 10586 is controlled by loci of the *mup* gene cluster with a size of 74, 417 bp (5). In this gene cluster, the *mupR*/*I* quorum sensing (QS) system is responsible for the transcriptional activation of the *mup* biosynthetic operons (6).

The LuxR family response regulator, MupR, plays a crucial role in the *mupR/I* QS system by sensing the *N*-acylhomoserine lactones (AHL) molecules produced by MupI and then activating the expression of *mupI* via a positive feedback loop and the expression of the *mup* operons at certain cell densities. Manipulation of the expression of the *mupR/I* QS genes as well as the stationary-phase sigma factor RpoS and other global regulators can enhance biosynthesis of mupirocin (7). Other regulators are also involved in regulating mupirocin biosynthesis, for example, the two-component RpeA/B system influences mupirocin biosynthesis via modulating QS, cell growth, and cell activities (8). The biosynthesis of multiple virulence factors or secondary metabolites in many *Pseudomonas* spp. is controlled by the two-component system (TCS) including GacS/A (9), CbrA/B (10), and RpeA/B (11). The molecular mechanisms of TCS regulation on the biosynthesis of mupirocin have still not been elucidated.

The Gac/Rsm signal transduction system globally controls the expression of numerous genes involved in multiple biosynthesis pathways of extracellular products through the Csr (Carbon storage repressor, called in *Escherichia coli*) or Rsm (Regulator of secondary metabolism, as designed in *Pseudomonas* spp.) system in Gammaproteobacteria (12). The Gac/Rsm system has been extensively investigated in *Pseudomonas* spp., and typically, the response is initiated with a membrane-located histidine kinase sensor GacS, which then transmits the signal to the response activator GacA in the cytoplasm. Upon interaction with environmental signals, GacS autophosphorylates and then phosphorylates its cognate GacA. The activated GacA protein then binds to the Gac-box (TGTAAGN_6_CTTACA as consensus) located in the promoter region of the small regulatory RNA (sRNAs, such as RsmX/Y/Z) loci and activates their expression. Subsequently, the sRNAs bind to RNA-binding repressors such as RsmA, which can interact with target mRNAs by recognizing the GGA trinucleotide motif (or CANGGAYG motif) in their single-stranded loop regions within the 5’-untranslated regions (5′-UTRs) and prevent protein synthesis transcriptionally and/or post-transcriptionally (13–16). There are one to four variants in the number of sRNAs across different species of *Pseudomonas*. For example, four sRNAs (RsmY, RsmZ, RsmV, and RsmW) were found to be organized in tandem in the genome of *P. aeruginosa* (17–19). RsmX, RsmY, and RsmZ are also found in *P. fluorescens, Pseudomonas protegens,* and *P. syringae,* respectively (20, 21), whereas only two sRNAs (RsmY and RsmZ) are present in *P. putida* (22). These sRNAs are GacA-dependent, and their presence and effects on gene expression vary among the different bacterial species. Likewise, some species encode different numbers of CsrA/RsmA orthologs, such as RsmE, RsmN, and RsmF in *P. aeruginosa* (23–25) and RsmE and RsmI in *P. fluorescens* (26). CsrA family proteins were first discovered as repressors of translation of target mRNAs, but recently, it has been demonstrated that CsrA family proteins can bind to different regions of mRNAs, exerting a negative or positive effect on their transcription, transcript stability, and/or translation process (27). In summary, the Gac/Rsm transduction cascade can affect the expression of numerous genes through GacA or CsrA protein binding to the target DNA or mRNA, respectively.

The Gac/Rsm system regulates the expression of several loci responsible for the biosynthesis of secondary metabolites (9). For example, lipase biosynthesis in *P. protegens* Pf-5 is positively regulated by the Gac-RsmE system via activation of *lipA* translation by RsmE (28), while biosynthesis of the antifungal lipopeptide massetolide A in *P. fluorescens* SS0101 is regulated by RsmY/Z and RsmA/E via a LuxR-type regulator (29). Furthermore, the biosynthetic operon of the antibiotic pyoluteorin (Plt) is also positively regulated by GacA and RsmA through a QS-mediated way in *P. aeruginosa* M18 (30). Moreover, the homologous *plt* gene cluster in *P. protegens* H78 is post-transcriptionally repressed by the Gac/Rsm-RsmE regulatory cascade but positively regulated by RsmA. Concurrently, a positive feedback loop driven by RsmA/E is required for Plt biosynthesis (31). For this reason, the production of Plt is enhanced by the deletion of repressor RsmE (32). In addition, *P. fluorescens* 2P24 produces 2,4-diacetylphloroglucinol (2,4-DAPG), which is an antifungal compound whose biosynthesis is affected by the Gac/Rsm system and the AHL-driven QS locus, *pcoR/I* (homologous to *mupR/I*) (33–35). The Gac/Rsm system activates the *phlACBD* gene cluster, which is required for the production of 2,4-DAPG, through the action of four sRNAs (RsmX/X1/Y/Z) sequestering RsmA and RsmE proteins that can bind to the 5′-UTR with a GGA motif of the *phlACBD* operon and then inhibit the *phlACBD* translation process in strain 2P24 (26).

The aim of the current work was to investigate the impact of regulatory elements of the Gac/Rsm transduction system on the expression of *mupR/I* genes and the biosynthesis of mupirocin, and its regulatory mechanism in *Pseudomonas* sp. NCIMB 10586. Two sRNAs of the Gac/Rsm system and five variants of the RsmA family protein were characterized and studied for their roles in mupirocin biosynthesis. Our study demonstrated that the MupR/I QS system and mupirocin biosynthesis were stringently controlled by GacS/A TCS and differentially affected by RsmA family proteins.

## RESULTS

### The GacS/A two-component system activates the biosynthesis of mupirocin in *Pseudomonas* sp. NCIMB 10586

**Fig 1:**
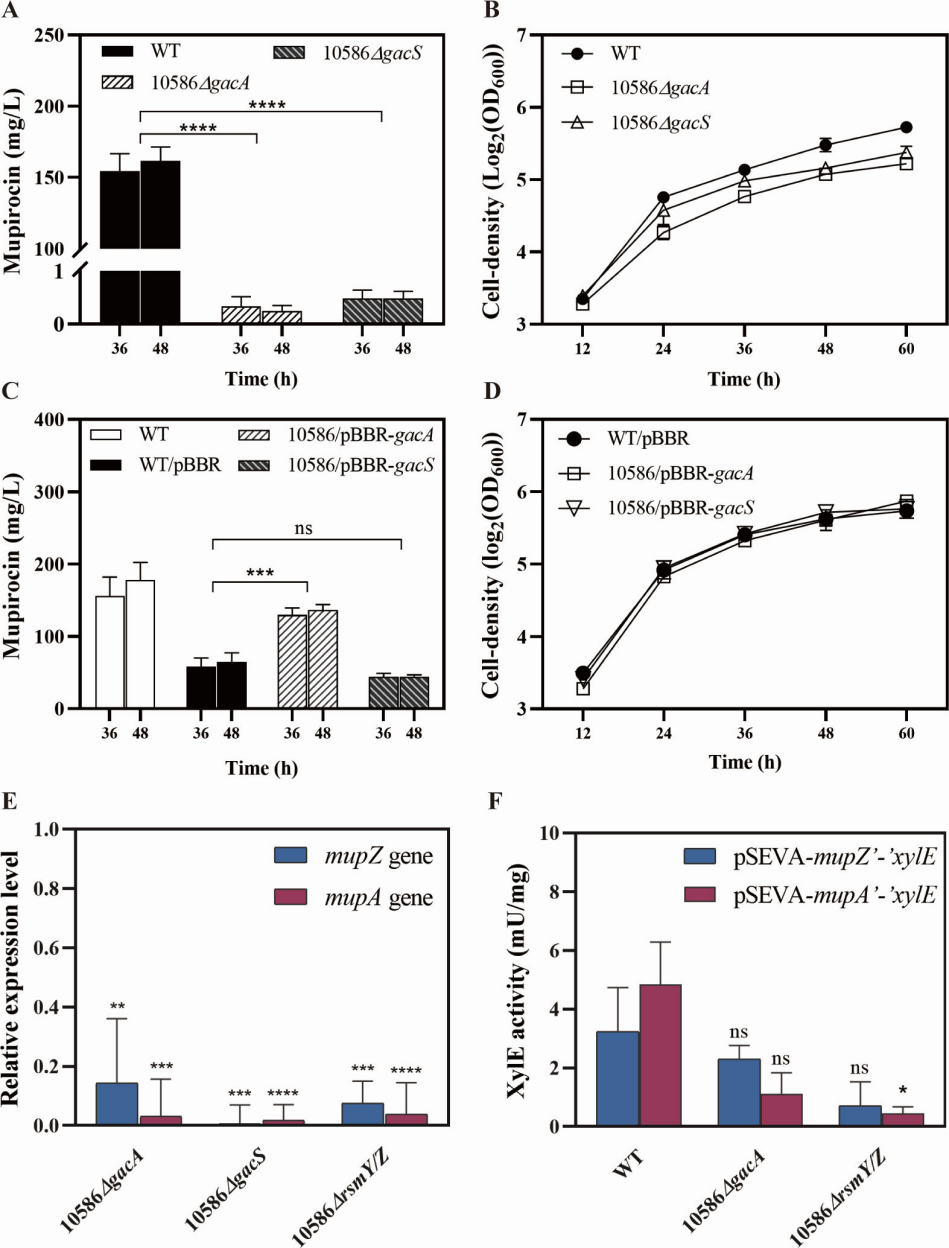
The influence of GacS/A TCS on the production of mupirocin, cell growth, and *mup* gene expression in *Pseudomonas* sp. NCIMB 10586. (**A–B**) Mupirocin production (**A**) and cell growth (**B**) in the *gacA* mutant, *gacS* mutant, and the wild-type grown on the MP medium. (**C–D**) Mupirocin production (**C**) and cell growth (**D**) of strain NCIMB 10586 carrying the *gacA* expression plasmid (pBBR-*gacA*), the *gacS* expression plasmid (pBBR-*gacS*), and the empty plasmid pBBR as the control in the MP medium. The production of mupirocin in strain NCIMB 10586 carrying the empty pBBR decreased compared to that of the plasmid-free wild-type strain. (**E**) Relative quantification of mRNA expression levels (fold change) of *mupZ* and *mupA* in the *gacA*, *gacS,* or *rsmY/Z* mutants compared to the respective gene expression in the wild-type strain. Significance of The expression levels are indicated in the column of the mutants. The 16S rRNA gene was set as a reference. (**F**) XylE activity of the *mupZ’-’xylE* and *mupA’-’xylE* transcriptional and translational dual reporters in the *gacA* mutant, *gacS* mutant, and the wild-type in the MP medium. The symbol “*” or “ns” indicated the activity differences from the reporter of the *gacA* mutant or the *rsmY/Z* mutant compared to that of the wild-type in the MP medium. Error bars show standard deviation among at least three repeats. “ns” indicates no significant differences, *** indicates *P* < 0.001, and **** indicates *P* < 0.0001.

The GacS/A TCS plays pivotal roles in regulating the biosynthesis of secondary metabolites in *Pseudomonas* sp. In order to investigate its regulatory role on mupirocin biosynthesis, a *gacA* knockout and a *gacS* knockout mutant of strain NCIMB 10586 were constructed as described in Materials and Methods and designated as 10586*△gacA* and 10586*△gacS*, respectively. Mupirocin production in strains 10586*△gacA* and 10586*△gacS* were both significantly reduced (Fig. 1A), and their growth curves were lower than that of the wild-type between 12 and 60 hours (Fig. 1B). The expression levels of the first two genes of the *mup* gene cluster were also tested in these *gac* mutant strains. The mRNA levels of *mupZ* and *mupA* in the *gac* mutants both notably decreased (Fig. 1E), which was consistent with the considerably lower amounts of mupirocin produced in the *gac* mutants. Furthermore, the *mupZ’-’xylE* and *mupA’-’xylE* transcriptional and translational dual reporter activities in the *gacA* mutant showed a decrease but no significant change compared to the wild-type (Fig. 1F). No GacA box-like sequence was found within the promoter regions of the *mup* biosynthetic gene cluster, suggesting that GacA might influence the mupirocin production indirectly by regulating the MupR/I QS system. To further elucidate the role of GacS/A in mupirocin biosynthesis, plasmids pBBR-*gacA* and pBBR-*gacS* were constructed and transformed into the wild-type strain to overexpress *gacA* and *gacS*, respectively. The overexpression of *gacA* significantly enhanced the production of mupirocin from 61.75 to 129.88 mg/L without affecting the growth of NCIMB 10586 (Fig. 1C and D). In addition, mupirocin production in the *gacA* knockout mutant was restored to wild-type levels by introducing the pBBR-*gacA* plasmid (Fig. S1). These results demonstrated that GacS/A TCS functions as an indirect activator in the expression of the *mup* gene cluster and the subsequent production of mupirocin.

### The GacS/A system positively regulates the *mupR/I* quorum sensing system

**Fig 2:**
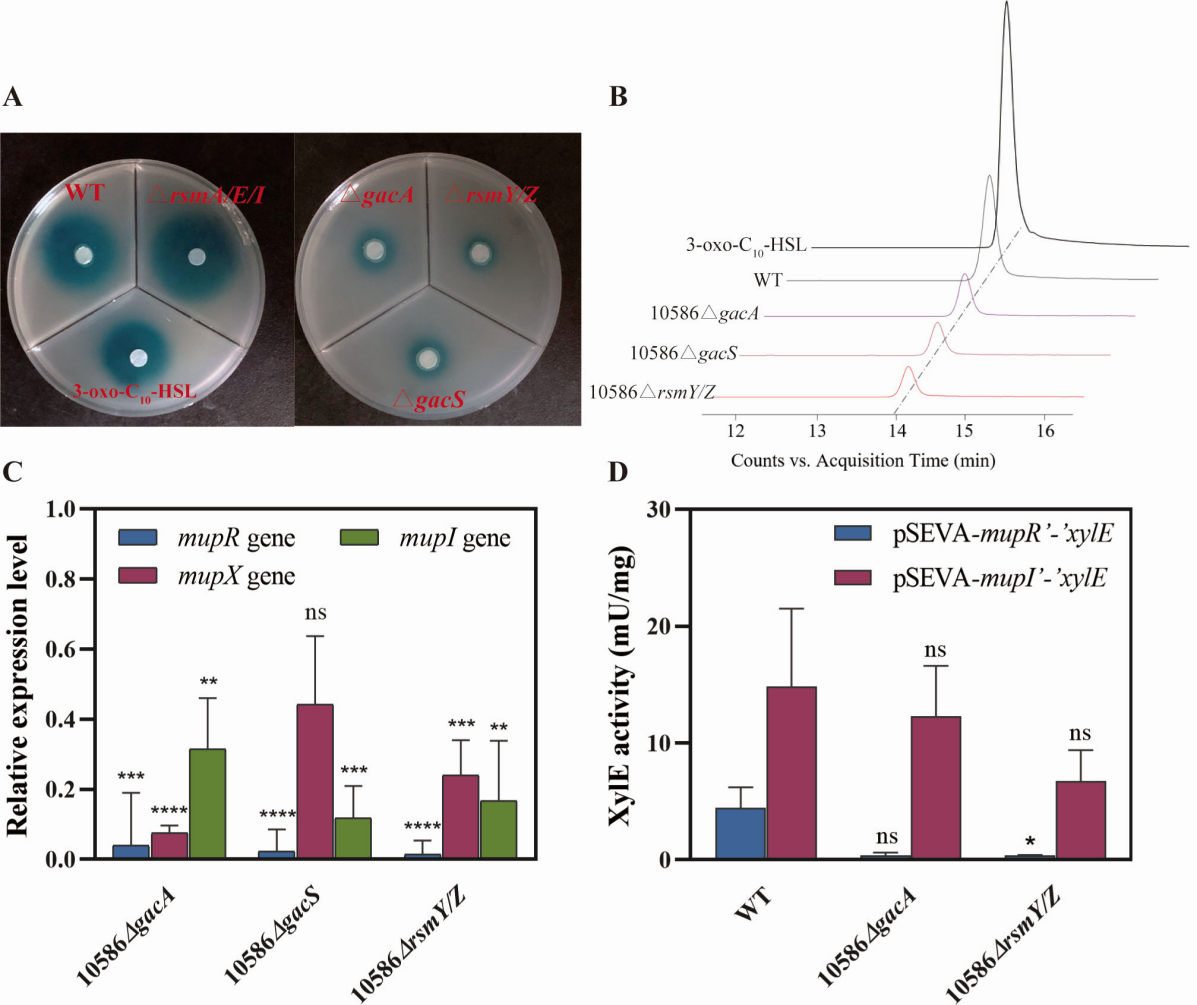
The AHL bioassay and the expression of QS genes in strains 10586*△gacA*, 10586*△gacS,* and 10586*△rsmY/Z*. (**A**) The AHL signal assay for strains 10586*△gacA*, 10586*△gacS*, 10586*△rsmY/Z*, and 10586*△rsmA/E/I*. The cognate signal of the *mupR/I* QS system, 3-oxo-C_10_-HSL, was set as the positive control. (**B**) Relative production levels of 3-oxo-C_10_-HSL by strains 10586*△gacA*, 10586*△gacS*, 10586*△rsmY/Z*, and the wild-type strain measured using HPLC-ESIMS analyses. Positive electrospray of extracts from the mutants and the wild-type revealed a peak corresponding to 3-oxo-C_10_-HSL. (**C**) Relative expression levels (fold change) of *mupR* and *mupI* in *gacA*, *gacS,* or *rsmY/Z* mutants with respect to gene expression levels in the wild-type strain. Significance of the expression levels is indicated in the column of the mutants. (**D**) XylE activity of the *mupR’-’xylE* and *mupI’-’xylE* transcriptional and translational reporters in strains 10586*△gacA*, 10586*△gacS*, 10586*△rsmY/Z*, and the wild-type strain. Significance of the expression levels is indicated in the activity column of the mutants. Error bars show standard deviation among at least three repeats. “ns” indicates no significant differences, * indicates *P* < 0.05, ** indicates *P* < 0.01, *** indicates *P* < 0.001, and **** indicates *P* < 0.0001.

Since previous studies have shown that mupirocin biosynthesis is regulated by the *mupR/I* genes and a few potential *lux* boxes were mapped upstream of *mupA* (6), it was important to determine possible cross-regulation of the GacS/A with the MupR/I QS system. We first confirmed that inactivation of both *mupR* and *mupI* led to the near disappearance of mupirocin production (Fig. S2). Determination of the QS signaling activity by an AHL bioassay evidenced that QS responses were severely damaged in the two mutants, 10586*△gacA* and 10586*△gacS* (Fig. 2A). The amount of the corresponding signal was subsequently confirmed by 3-oxo-C_10_-HSL accumulation from HPLC-ESIMS analysis (Fig. 2B; Fig. S3), which showed that the signaling amount was decreased by almost 70% in the *gac* mutants. We also investigated the mRNA level of *mupR* and *mupI* as well as the *mupR’-’xylE* and *mupI’-’xylE* transcriptional and translational dual reporter activity (Fig. S4) in the *gacA* or *gacS* mutant. The results revealed that the expression levels of *mupR* and *mupI* decreased significantly (Fig. 2C) and the activities of the *mupR’-’xylE* reporter declined in the *gacA* mutant, but the *mupI’-’xylE* reporter displayed no significant differences in the enzymatic activity levels between the *gacA* mutant and the wild-type (Fig. 2D). These results clearly indicated that GacS/A controlled the biosynthesis of mupirocin via the regulation of the MupR/I QS system in NCIMB 10586.

### Characterization of GacA-dependent small regulatory RNAs in *Pseudomonas* sp. NCIMB 10586

**Fig 3:**
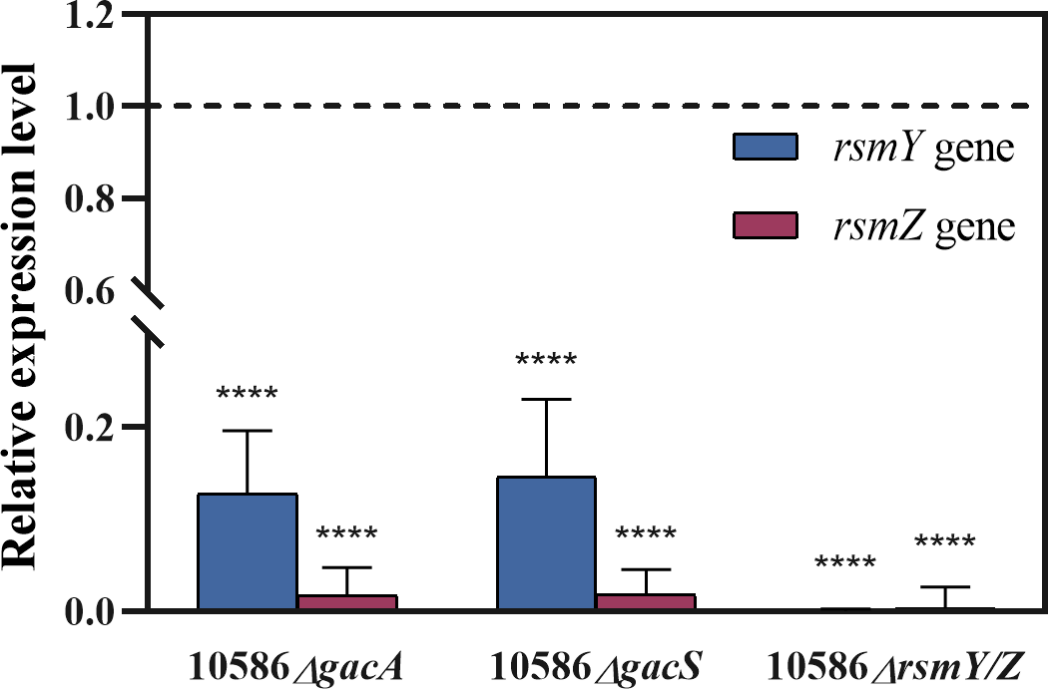
The effects of *gacA* or *gacS* inactivation on the expression of small regulatory RNAs RsmY and RsmZ in *Pseudomonas* sp. NCIMB 10586. Relative expression levels of *rsmY* and *rsmZ* in the *gacA* and *gacS* mutants with reference to The respective gene expression levels in the wild-type strain. The RsmY and RsmZ expression levels in the *rsmY/Z* double mutant are also indicated. Significance of the expression levels is indicated in the column of the mutants. Error bars show standard deviation among at least three repeats. *** indicates *P* < 0.001, and **** indicates *P* < 0.0001.

The GacS/A TCS system mainly plays a major role in complex regulatory cascades or networks, where the response regulator GacA directly modulates the transcription of A-T rich small RNAs and other regulatory factors. In order to identify the archetypical Rsm-type regulatory RNAs in *Pseudomonas* sp. NCIMB 10586, a BLASTN search was performed to assess the presence of homologs within its genome (GenBank accession number LR027557). Two Rsm-type regulatory sRNAs were found, exhibiting 96.61% and 90.15% identity to RsmY and RsmZ of *P. protegens* CHA0, respectively. In addition, conserved GacA-boxes were predicted within their promoter regions (Fig. S5). The expression levels of the RsmY and RsmZ assayed by qRT-PCR were significantly decreased in both mutant strains (10586*△gacA* and 10586*△gacS*) compared to the wild-type level (Fig. 3), suggesting that their expression requires the activation of GacA.

### Influences of RsmY and RsmZ on the biosynthesis of mupirocin and on the *mupR/I* QS system

**Fig 4:**
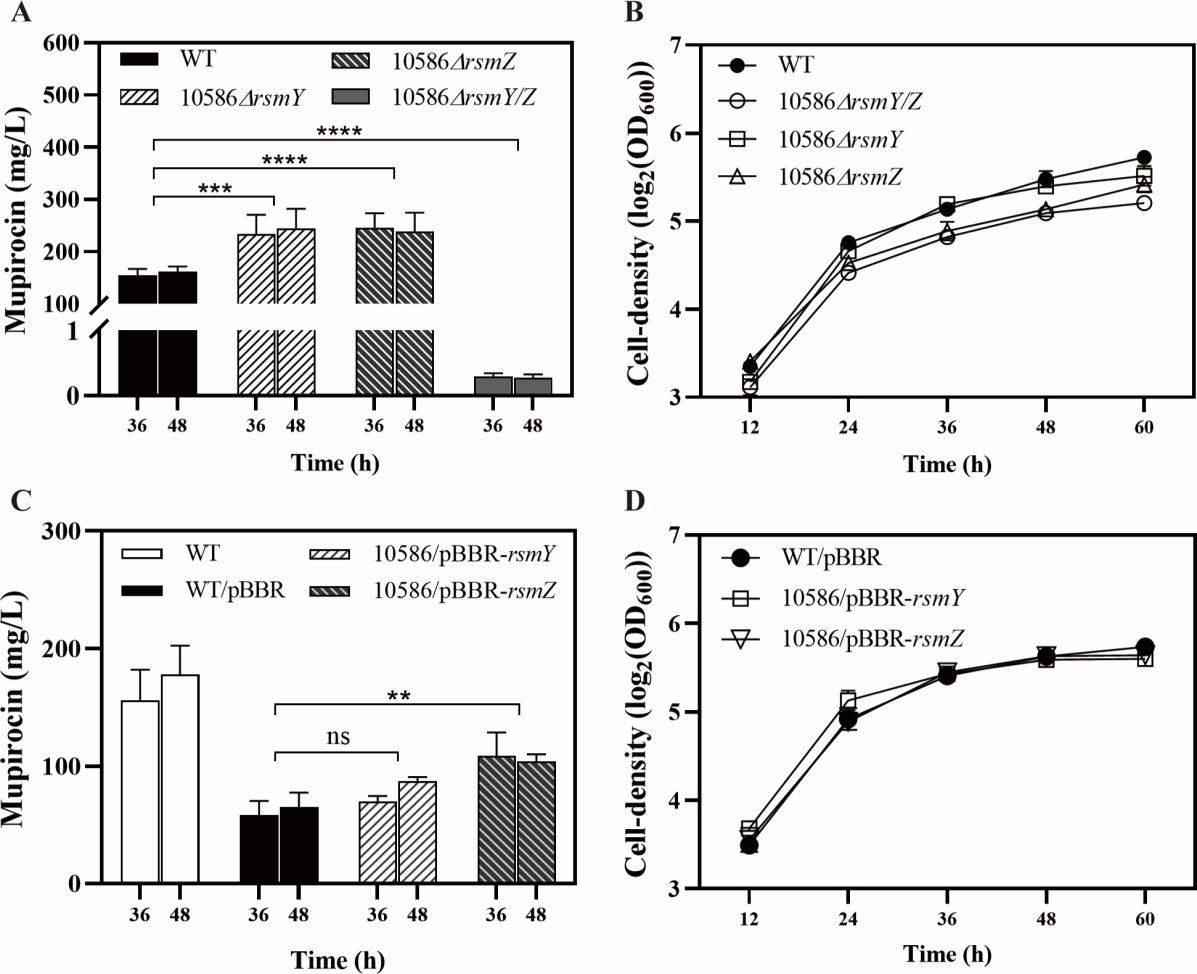
The effects of small regulatory RNAs RsmY/Z on the production of mupirocin and cell growth of *Pseudomonas* sp. NCIMB 10586. (**A–B**) Mupirocin production (**A**) and cell growth (**B**) of the *rsmY* mutant, *rsmZ* mutant, *rsmY/Z* double mutant, and the wild-type in the MP medium. (**C–D**) Mupirocin production (**C**) and cell growth (**D**) of strain NCIMB 10586 carrying the *rsmY* expression plasmid (pBBR-*rsmY*), the *rsmZ* expression plasmid (pBBR-*rsmZ*), and the empty plasmid pBBR as the control in the MP medium. The production of mupirocin in strain NCIMB 10586 carrying the empty pBBR decreased compared to that of the plasmid-free wild-type strain. Error bars show standard deviation among at least three repeats. “ns” indicates no significant differences, ** indicates *P* < 0.01, *** indicates *P* < 0.001, and **** indicates *P* < 0.0001.

To investigate the effects of Rsm-type regulatory RNAs on the biosynthesis of mupirocin, single and double mutants of RsmY and RsmZ were generated. The production of mupirocin was increased to 244.84 ± 36.87 mg/L in the *rsmY* mutant and 237.86 ± 36.32 mg/L in the *rsmZ* mutant compared to the amount of 164.98 mg/L in the wild-type. However, mupirocin production decreased significantly in the *rsmY/Z* double mutant, indicating the significant activation roles of RsmY and RsmZ on the biosynthesis of mupirocin (Fig. 4A). Regarding cell growth of the mutants, the curve of 10586*△rsmY/Z* was lower than that of the wild-type (Fig. 4B). Both of these phenotypes are in accordance with the results obtained with *gac* mutants (Fig. 1). The expression levels of *mupZ*, *mupA*, *mupR*, *mupX,* and *mupI* were noticeably reduced in the double mutant 10586*△rsmY/Z* (Fig. 1E and 2C). In addition, the enzymatic activities of *mupA’-’xylE* and *mupR’-’xylE* reporters decreased in the *rsmY/Z* mutant (Fig. 1F and 2D). These results indicated that the RsmY and RsmZ sRNAs in *Pseudomonas* sp. NCIMB 10586, as necessary participants in the GacS/A signal transduction system, affected mupirocin biosynthesis and the *mupR/I* QS system possibly at transcription and posttranscriptional levels.

### RsmZ played a more significant role in the mupirocin biosynthesis and the expression of *mupR/I* compared to RsmY

**Fig 5:**
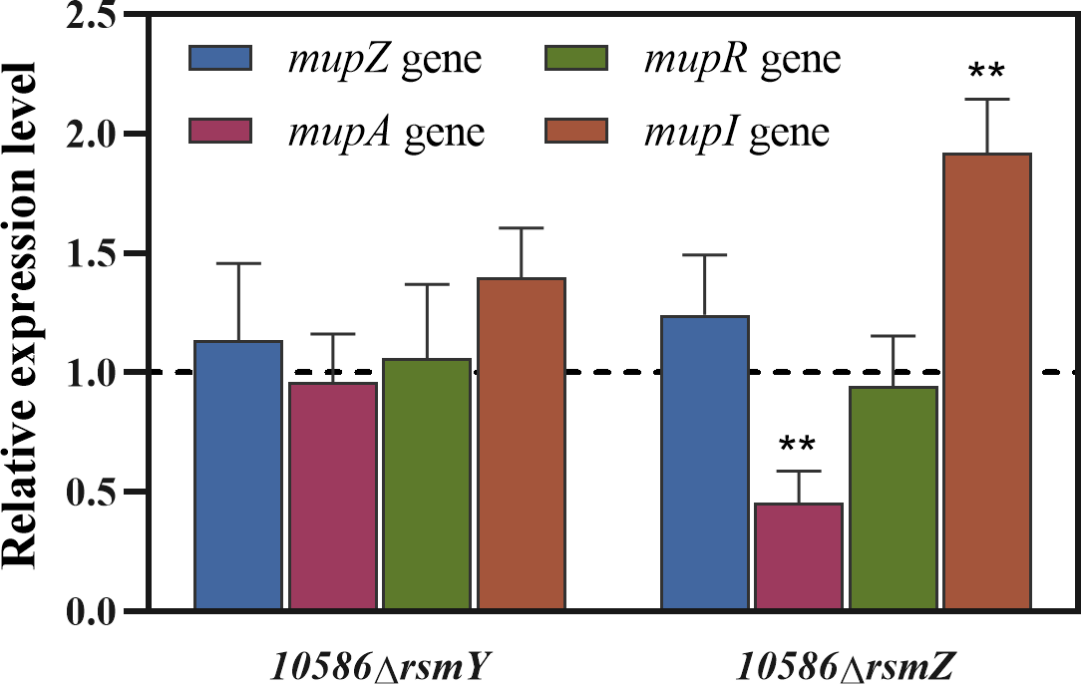
The impacts of *rsmY* and *rsmZ* on expression levels of the *mup* genes. The relative expression levels of *mupZ*, *mupA*, *mupR*, and *mupI* in the *rsmY* and *rsmZ* mutants with reference to the gene expression levels of the wild-type strain. Significance of the expression levels is indicated in the column of the mutants. The error bars show standard deviation among at least three repeats. “ns” indicates no significant differences, and ** indicates *P* < 0.01.

In the Gac/Rsm regulatory system, small regulatory RNAs with their conservative secondary structure sequester Rsm-type proteins and eliminate the binding of Rsm proteins to the target mRNAs, thereby affecting the transcription, RNA stability, and the translation initiation process of mRNAs (9). In this study, the mRNA levels of the *mupZ*, *mupA*, *mupR,* and *mupI* were tested in strains 10586*△rsmY and* 10586*△rsmZ*. Results indicated a significant increase in *mupI* mRNA and a decrease of *mupA* mRNA in strain 10586*△rsmZ* compared to those in the wild-type (Fig. 5). In contrast, their expression showed no obvious difference between 10586*△rsmY* and the wild-type strain. As illustrated in Fig. 4B, the cell-growth of the *rsmZ* mutant showed a greater decrease compared to both the wild-type and the *rsmY* mutant. Additionally, the overexpression of *rsmZ* in a plasmid enhanced the production of mupirocin (Fig. 4C). These results highlighted that RsmZ exhibited an important role with respect to cell growth, mupirocin biosynthesis, and the related gene expression in NCIMB 10586.

### Identification of CsrA family proteins in NCIMB 10586

**Fig 6:**
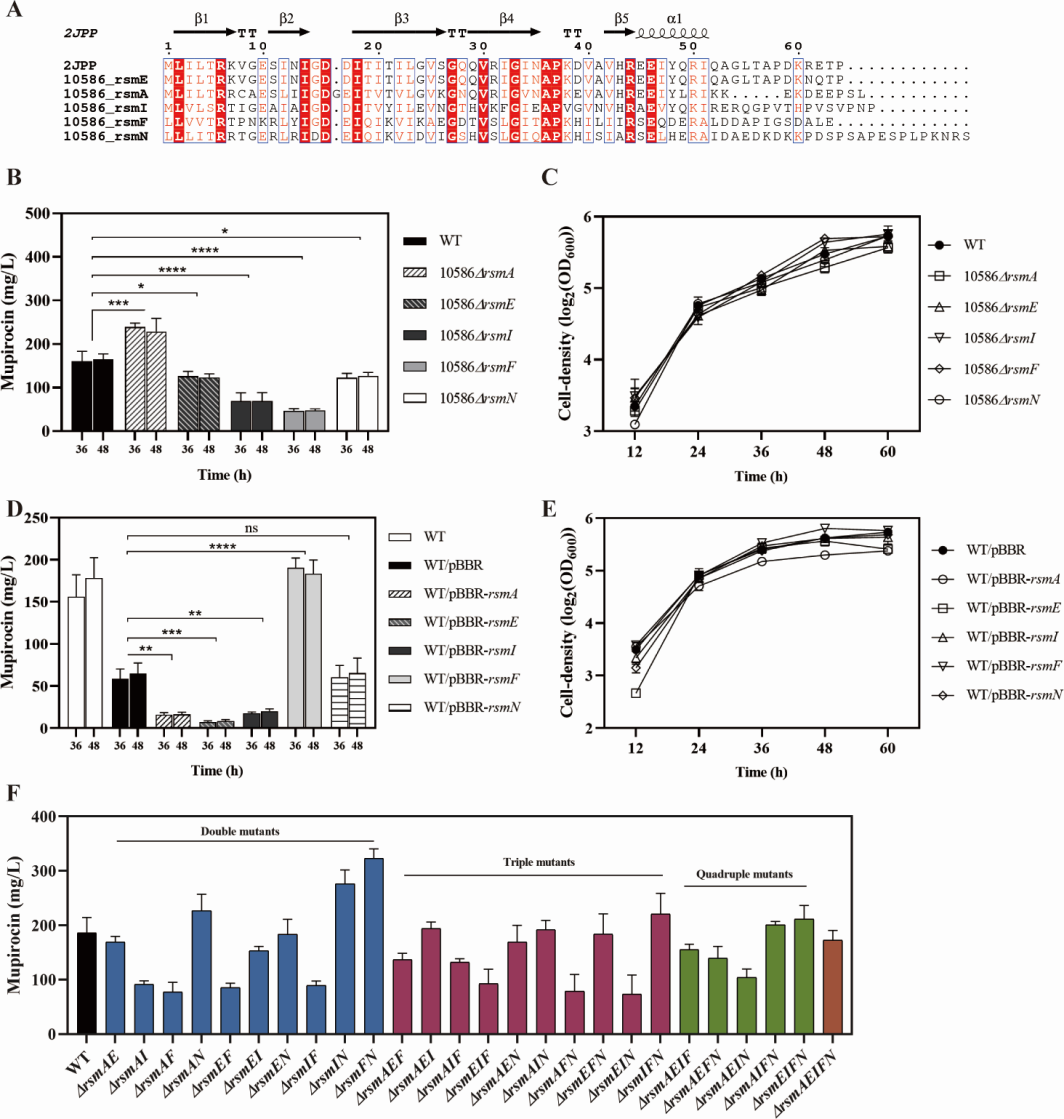
The identification and effects of Rsm-type proteins in *Pseudomonas* sp. NCIMB 10586. (**A**) Multiple alignment of amino acid sequences of RsmA family proteins with a well-characterized RsmE of P. (2JPP). (**B–C**) Mupirocin production (**B**) and cell growth (**C**) of the *rsmA* mutant, *rsmE* mutant, *rsmI* mutant*, rsmF* mutant, *rsmN* mutant, and the wild-type in the MP medium. (**D–E**) Mupirocin production (**D**) and cell growth (**E**) of strain NCIMB 10586 carrying the *rsmA* expression plasmid (pBBR-*rsmA*), the *rsmE* expression plasmid (pBBR-*rsmE*), the *rsmI* expression plasmid (pBBR-*rsmI*), the *rsmF* expression plasmid (pBBR-*rsmF*), the *rsmN* expression plasmid (pBBR-*rsmN*), and the empty plasmid pBBR as the control in the MP medium. The production of mupirocin in strain NCIMB 10586 carrying the empty pBBR decreased compared to that of the plasmid-free wild-type strain. (**F**) Mupirocin production by the double, triple, quadruple, and quintuple mutants of *rsmA* family genes. Error bars show standard deviation among at least three repeats. “ns” indicates no significant differences, * indicates *P* < 0.05, ** indicates *P* < 0.01, *** indicates *P* < 0.001, and **** indicates *P* < 0.0001.

CsrA family proteins play an important role in regulating the biosynthesis of secondary metabolites. In most cases, their homologs are called RsmA-type proteins in *Pseudomonas* and regulate phenotypes in a comparable manner. Here, five homologs of CsrA, i.e., RsmA, RsmE, RsmI, RsmF, and RsmN, were identified in *Pseudomonas* sp. NCIMB 10586 via a BLASTP analysis. RsmA, RsmE, and RsmI share the highest similarity of 100%, 100%, and 83.82% to the ortholog proteins RsmA, RsmE, and CsrA in *P. fluorescens* A506, respectively. For RsmF and RsmN, they share 68.05% and 58.5% similarities with the CsrA protein of *Pseudomonas synxantha* R2-4-08W, respectively. In addition, RsmA and RsmE share the similarity of 80.33% and 67.19% to the well-characterized RsmA in *P. aeruginosa* PAO1, respectively. Further, RsmI shares 51.92% similarity with RsmI in *P. putida* KT2440. These identified Rsm proteins in strain NCIMB 10586 exhibit the conserved RNA-binding domain (Fig. 6A) by aligning with the well-established RsmE (PDB ENTRY ID: J2PP) of *P. protegens* CHA0 (23).

### Pleiotropic effects of Rsm-type proteins on the biosynthesis of mupirocin

Single deletion of *rsmA*, *rsmE*, *rsmI*, *rsmF*, and *rsmN* differentially affected mupirocin biosynthesis in strain NCIMB 10586. Inactivation of *rsmA* enhanced mupirocin production, whereas the mupirocin yields in strains 10586*△rsmE,* 10586*△rsmI,* 10586*△rsmF,* and 10586*△rsmN* all decreased (Fig. 6B and C). Overexpression of *rsmA*, *rsmE,* or *rsmI* downregulated mupirocin biosynthesis. In contrast, *rsmF* overexpression led to a threefold increase in the yield of mupirocin compared to that in the wild-type (Fig. 6D and E). These results indicated that the genetic manipulation of the genes encoding Rsm proteins can affect the biosynthesis of mupirocin. In order to investigate the regulatory roles of Rsm proteins on mupirocin biosynthesis, the double, triple, quadruple, and quintuple mutants of the *rsm* loci were constructed (Table S1). However, no significant variation in mupirocin production was found between these *rsm* mutants and the wild-type, whereas three strains (10586*△rsmAEI*, 10586*△rsmAEIN*, and 10586*△rsmAEIFN*) exhibited similar levels of mupirocin production compared to that in the wild-type. An increase in mupirocin production (322.73 ± 17.69 mg/L) was induced by the inactivation of both *rsmF* and *rsmN* (Fig. 6F). These results indicated the Rsm-type proteins have pleiotropic effects on mupirocin biosynthesis and implied that other regulators may be involved in the Gac/Rsm cascade.

### Identification of Rsm protein target sites in *mupR* and *mupI* mRNA leaders and the effects of *gacA* and *rsmY/Z* inactivation on the post-transcriptional expression levels of *mupR* and *mupI*

**Fig 7:**
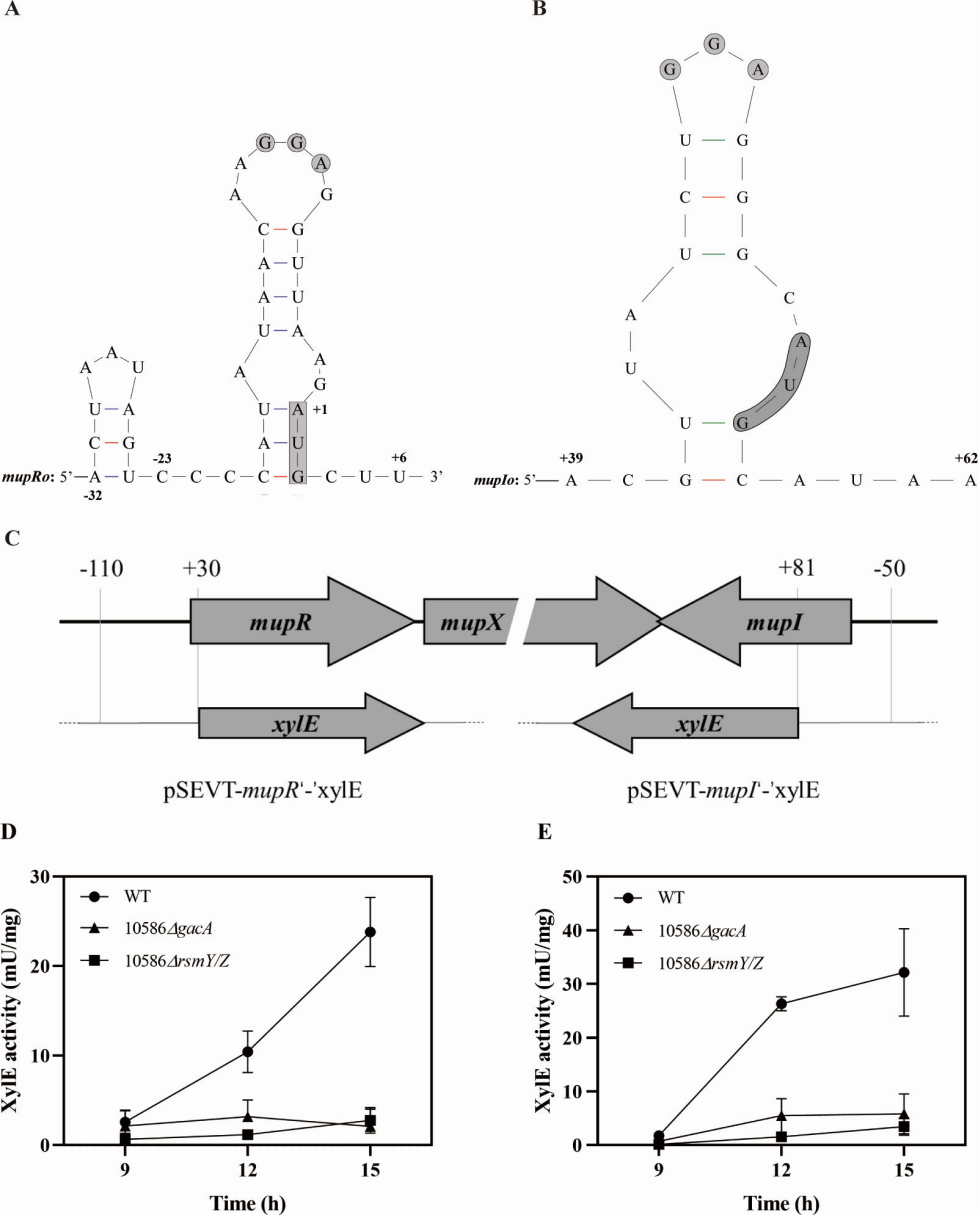
The influences of *gacA* and *rsmY/Z* mutation on the translational level of *mupR* and *mupI*. (**A–B**) Predicted secondary structure of the mRNA leader sequence near the translational start site of *mupR* (**A**) and *mupI* (**B**) by using mFold server. The conserved GGA motifs are marked with gray-filled circles. Arabic numbers in the proximity of nucleotides represent the position relative to the AUG start codon. (**C**) Construction map of the translational reporters, the *mupR’-’xylE* (left) and *mupI’-’xylE* (right) reporters. (**D–E**) The expression levels of *mupR* (**D**) and *mupI* (**E**) in 10586*△gacA* and 10586*△rsmY/Z* compared to expression levels in the wild-type strain.

CsrA family proteins recognize and bind to a conserved RNA-motif featured with a stem-loop, thereby affecting the post-transcriptional process of target mRNAs. The presence of the GGA motif close to the leading sequence of mRNAs is pivotal for translational repression by RsmA family proteins. In this study, the secondary structures formed by the mRNA leaders of *mupR* and *mupI* were mapped (Fig. 7A and B). The leading sequence of *mupR* mRNA was found to contain a conserved “CANGGANG” motif overlap with the RBS and the imperfect GGA motif located downstream the transcriptional start site of *mupI* (+48 to +50 nt, relative to the AUG start codon). In order to investigate the possible role of CsrA family proteins, two post-transcriptional reporters, pSEVT-*mupRo’-’xylE* and pSEVT-*mupIo’-’xylE*, were constructed and transferred into 10586*△gacA* and 10586*△rsmY/Z* (Fig. 7C). Results demonstrated that the post-transcriptional levels of *mupR* and *mupI* in both mutants were decreased compared with those of the wild-type (Fig. 7D and E). These results indicated that GacA and RsmY/Z played a role in the biosynthesis of mupirocin, most probably via regulation at the post-transcriptional levels of the QS genes, *mupR* and *mupI*.

### RsmF protein interacts with the 5’-untranslated region of *mupR* mRNA

**Fig 8:**
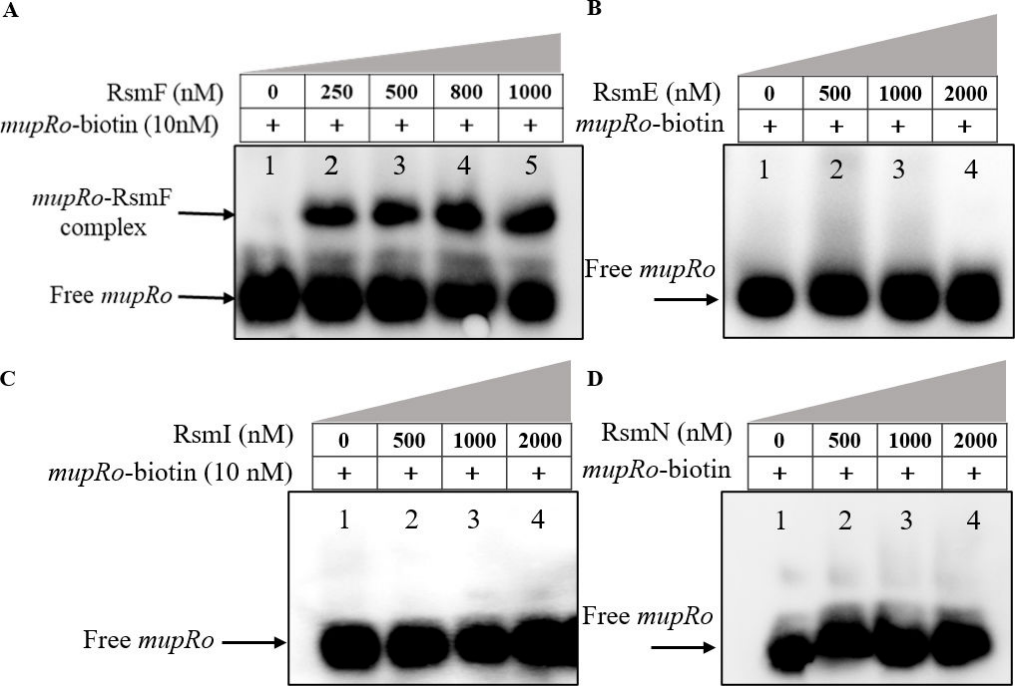
The interactions of RsmA family proteins with the *mupR* mRNA 5’ leader. (**A**) Direct interaction of RsmF with the *mupR* mRNA 5’ leader. Lane 1, negative control, contains only the genetic material. Lanes 2–5, 10 nM biotin-labeled *mupRo* was incubated in a concentration gradient (250 nM to 1,000 nM) with purified RsmF protein. The arrow of free *mupRo*-RsmF complex represents the shift bands of the complex of RsmF and biotin-labeled *mupR* mRNA 5’leader. (**B–D**) No interaction was discovered between *mupR* mRNA 5’ leader with RsmE (**B**), RsmI (**C**), and RsmN (**D**), respectively. Lane 1, negative control, contains only the genetic material. Lanes 2–4, 10 nM biotin-labeled *mupRo* was incubated in a concentration gradient (500 nM to 2,000 nM) with respective purified Rsm proteins. The “Free *mupRo*” arrow represents the bands of biotin-labeled *mupR* mRNA 5’leader.

The leader region of *mupR* mRNA possesses a conserved “AAGGA” motif within the RBS (Fig. 7A), and in contrast, the mRNA leading sequence of *mupI* contains an imperfect motif downstream of its start codon. This implied that the *mupR* mRNA leader is a likely target of RsmA family proteins. The mRNA leader sequence (−31 to +6 nt, relative to AUG start codon) with a biotin label of *mupR* was synthesized to perform an electrophoretic mobility shift assay (EMSA) with the purified histidine-tagged RsmF protein. As shown in Fig. 8A, the shifted bands representing the RsmF-*mupRo* complex indicated that RsmF had the ability to bind to the 5’UTR of *mupR* mRNA at a concentration range of 250 nM to 1 µM. In addition, RsmE, RsmI, and RsmN proteins were also purified and subjected to EMSA with the *mupRo* probe. However, no shifted RNA–protein complex band was observed (Fig. 8B-D). Our results demonstrated that the overexpression of RsmF led to an increase in mupirocin production (Fig. 6D); it was shown that RsmF interacted with the *mupR* 5’ leader (Fig. 8A). It was therefore hypothesized that RsmF enhanced the expression of MupR (as the activator of the *mup* gene cluster) at a post-transcriptional level by binding to the 5’UTR of *mupR* mRNA. However, the potential molecular mechanisms underlying the positive regulation of *mupR* expression by RsmF remain unknown.

## DISCUSSION

**Fig 9:**
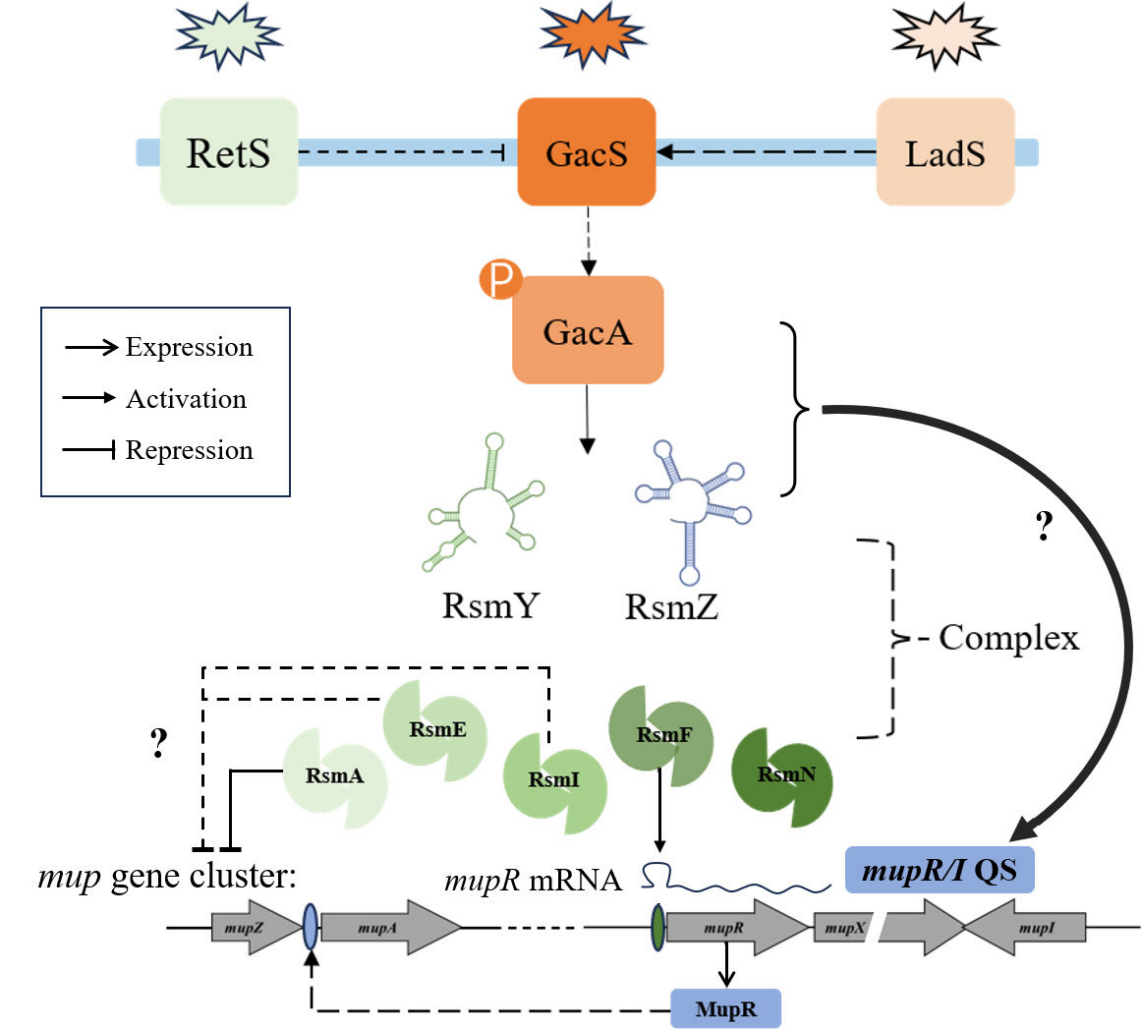
Proposed regulatory model of the Gac/Rsm transduction system on mupirocin biosynthesis in *Pseudomonas* sp. NCIMB 10586. Bold arrows, positive regulations; bold lines with a flattened end, negative regulations; thin arrows, biogenesis and transport; Question marks, uncharacterized mechanisms. The Gac/Rsm system regulates mupirocin biosynthesis via GacA-RsmY/Z-*mupR/I* QS system cascade, involving two key pathways: (1) RsmF protein positively regulates mupirocin biosynthesis by interacting with *mupR* mRNA 5’ leader (2). GacS/A two-component system positively regulates mupirocin biosynthesis through sRNAs (RsmY and RsmZ) sequestering RsmA protein via unknown molecular mechanisms. Other RsmA homologous proteins, RsmE, RsmI, and RsmN, showed unclear effects in regulating mupirocin biosynthesis.

In *Pseudomonas* species, the global Gac/Rsm signal transduction system regulates the gene expression of multiple pathways such as virulence formation, secondary metabolite production, and quorum sensing in response to environmental signals (21, 36, 37). This study investigated the role of the Gac/Rsm cascade on the biosynthesis of mupirocin, a secondary metabolite, and the expression of *mupR/I* QS system in the mupirocin-producing model strain *Pseudomonas* sp. NCIMB 10586. Our findings reveal that the Gac/Rsm system positively regulates mupirocin biosynthesis, mainly through regulation of the *mupR/I* QS system. Based on these results, we propose a regulatory model of Gac/Rsm cascade in mupirocin biosynthesis (Fig. 9) involving two key pathways: (1) RsmF protein directly interacts with 5’ leader of *mupR* mRNA, positively regulating mupirocin biosynthesis, and (2) GacS/A TCS positively regulates mupirocin biosynthesis through sRNAs sequestering RsmA protein, and RsmA negatively regulates mupirocin biosynthesis via unknown molecular mechanisms, as evidenced by increased mupirocin production in the *rsmA* mutant and decreased mupirocin production in the strain of *rsmA* overexpression (10586/pBBR-*rsmA*) (Fig. 6). Two GacA-dependent small regulatory RNAs, RsmY and RsmZ, are crucial in the regulatory cascade of mupirocin biosynthesis, which can be reflected by the results that the *rsmY/Z* double mutant led to a significant decline in the expressions of *mup* genes (*mupZ*, *mupA,* and QS genes), post-transcriptional levels of *mupR/I* system, QS signal accumulation, and mupirocin production (Fig. 1, 2 and 7). Other RsmA homologous proteins, RsmE, RsmI, and RsmN, showed unclear effects in regulating mupirocin biosynthesis, as determined by single and multiple knockout mutants. The pleiotropic effects of Rsm-type proteins on the biosynthesis of mupirocin implied complicated regulatory mechanisms behind the impact of the global Gac/Rsm system on mupirocin biosynthesis. At the top of the Gac/Rsm cascades, the GacS kinase phosphorylates itself and then the GacA protein by sensing unknown extracellular signals. The phosphorylation process of the GacA protein can also be affected by two important sensor kinases, RetS and LadS (38, 39), whose effects on mupirocin biosynthesis were further tested to further illustrate the schematic model. The *retS* mutant showed an increase in mupirocin production, while the *ladS* mutant sustained a mupirocin production similar to the wild-type (Fig. S6). This aligns with those of previous studies, where RetS was found to inhibit GacS activity by reducing phosphotransfer to GacA (40), whereas LadS activates GacS/A in response to calcium signals (41).

The *gacA*, *gacS*, or *rsmY/Z* mutant strains displayed significant reduction in AHL accumulation (Fig. 2A and B) and in both transcriptional (Fig. 1 and 2) and posttranscriptional levels (Fig. 7) of *mupR/I* genes. These results signify that the *mupR/I* QS system is stringently regulated by the Gac/Rsm regulatory cascade and in turn regulates mupirocin biosynthesis. Our findings align with previous research in which the GacS/A system positively regulates the biosynthesis of secondary metabolites including pyrrolnitrin, pyoluteorin, and phenazine through modulating the AHL-mediated QS systems in *Pseudomonas* species (31, 42–45). Furthermore, it is deduced that the RsmA protein binds to the *mupR* mRNA leader to inhibit its expression, a repression that can be alleviated through competitive sequestration by sRNAs (Fig. 9). Unfortunately, in our study, the soluble form of RsmA was too unstable to conduct EMSA experiments; thus, its possible interaction with *mupR* 5’ leader could not be determined. In contrast, RsmF, positively regulates the biosynthesis of mupirocin (Fig. 6B and D) and shows the ability to bind to the 5’UTR of *mupR* (Fig. 8A). The results indicate that the interaction of RsmF with *mupR* mRNA promotes *mupR* expression post-transcriptionally, thereby enhancing the expression of the *mup* gene cluster (Fig. 9). The observed mupirocin production in strains with *rsmE* or *rsmI* expression plasmids suggests that RsmE/I could be an inhibitor for mupirocin biosynthesis. However, the production in *rsmE* and *rsmI* mutants, together with the multiple mutants of *rsmA/E/I,* revealed the unclear regulatory roles of RsmE/I on mupirocin biosynthesis (Fig. 6). The pleiotropic effects of these RsmA family proteins on mupirocin biosynthesis can also be evidenced by the results in the multiple mutants of the five identified *rsmA* family genes (Fig. 6F).

In *Pseudomonas* sp. NCIMB 10586, RsmZ exhibited a more evident GacA dependency than RsmY, as indicated by the more significant reduction in RsmZ expression in 10586*△gacA* (Fig. 3). This difference could be due to variations in the conserved yet diverse palindromic structures of the predicted GacA-binding sites within the 5’UTRs of RsmY and RsmZ (Fig. S4). However, in addition to GacS/A, the expression of RsmY/Z can be affected by other TCS, such as AlgZ/R, as reported in several studies, which cannot be excluded in terms of their regulatory roles on sRNAs (46–48). Herein, either *rsmY* or *rsmZ* mutants and overexpression of *rsmZ* led to an increase in the production of mupirocin. Also, some targets have been provided by multiple mutants to promote mupirocin biosynthesis, such as the *rsmF/N* double deletion, whose resulting production was up to 322.73 ± 17.69 mg/L. These results imply that the genetic manipulation of Gac/Rsm global regulators is an alternative strategy to promote mupirocin biosynthesis.

In conclusion, our study revealed that the mupirocin biosynthesis and *mupR/I* QS system are regulated by the global Gac/Rsm transduction system via either RsmF directly binding to the 5’ UTR of *mupR* or via a GacA-RsmY/Z-RsmA regulatory cascade in targeting the *mup* gene cluster through unknown molecular mechanisms. The findings provide insights into the regulatory role of the Gac/Rsm system on mupirocin biosynthesis and the *mupR/I* QS system, thereby establishing a foundation for further improvements in mupirocin production.

## MATERIALS AND METHODS

### Bacteria, vectors, oligonucleotides, and growth condition

The bacterial strains and plasmids in this study are described in Table S1, and the oligonucleotide primers are listed in Table S2. All mutant strains and the wild-type derived from *Pseudomonas* sp. NCIMB 10586 were incubated at 28°C in Luria–Bertani (LB) as seed broth and cultured in 250-mL concaved Erlenmeyer flasks containing 60 mL mupirocin production (MP) medium (30 g/L tryptone, 50 g/L glycerol, 10 g/L glucose, 5 g/L NaCl, 5 g/L, 5 g/L corn steep powder, 0.5 g/L NH_4_Cl, and 0.5 g/L (NH_4_)_2_SO_4_), as described by Huang et al. (8). *Agrobacterium tumefaciens* NTL4 (pZLR4), *E. coli* DH5*α*, and S17-1 were cultured at 37°C in LB medium. Antibiotics were used at the following working concentrations (μg/mL): ampicillin (Amp) 100, kanamycin (Km) 50, and gentamycin (Gm) 15 for both strain NCIMB 10586 and *E. coli*. The following compounds were applied when necessary: 20 µg/mL X-Gal in blue/white selection, 20% sucrose in counter-selection of the suicide vector pK18mob*sacB*, and 0.5 µg/mL IPTG in promoter induction.

### DNA manipulation

All molecular biological operations not described in detail were conducted according to standard procedures (49). All restriction enzymes, PrimeSTAR polymerases, DNA In-Fusion HD Cloning kits and genomic extraction kit were purchased from Takara (Takara Bio Inc., Shiga, Japan) and were used according to the manufacturers’ specifications. The reagent kits for plasmid and DNA recovery from the gels and fragment purification were purchased from Magen Biotechnology Co., Ltd (Guangzhou). Primer synthesis and DNA sequencing were carried out by Shanghai Saiheng Biotechnology Co., Ltd.

### Construction of the gene knockout mutants

The mutants of *Pseudomonas* sp. NCIMB 10586 are listed in Table S1. The in-frame deletion or inactivation of *gacA*, *gacS*, *rsmY*, *rsmZ*, *rsmA*, *rsmE*, *rsmI*, *rsmF,* and *rsmN* genes was conducted using a non-scar homologous recombination method described by Huang et al. (8). The upstream and downstream flanking sequences of candidate genes were amplified by PCR from the genome of strain NCIMB 10586. The corresponding fragments were ligated with the pK18mob*sacB* (predigested with *Eco*R I and *Hind* III) using In-Fusion assembly. The recombinant plasmids were introduced into *E. coli* S17-1 (*λ*pir) competent cells and then integrated within target genes in the wild-type or mutants of strain NCIMB 10586 via conjugation. The mutant strains were verified through colony PCR and confirmed by DNA sequencing. The double mutation of *rsmY* and *rsmZ* along with the double, triple, quadruple, and quintuple mutations of *rsmA/rsmE/rsmI/rsmF/rsmN* were conducted under the same method by introducing into the relative mutants.

### Complementation and overexpression

To evaluate the impact of Gac/Rsm regulatory genes on mupirocin production, the expression plasmids for complementation and overexpression of these genes were constructed. The coding regions of these candidate genes were amplified and separately inserted into the pBBR1MCS-2 vector downstream of the *lac* promoter by using the in-fusion cloning method (Takara Clontech). For example, the coding region of *gacA* was amplified with 5’ and 3’ homology arms and then inserted into the amplified and linearized pBBR1MCS-2 with complementary DNA ends, generating the *gacA* expression vector (pBBR-*gacA*). Similarly, seven expression plasmids of *rsmY*, *rsmZ*, and *rsmA/E/I/F/N* genes were constructed in the pBBR1MCS-2 vector, respectively (Table S1). In these recombinant expression plasmids, the plasmid *lac* promoter was used to drive the expression of the inserted genes. The constructed plasmids were then separately introduced into the respective mutants as complementation plasmids and placed in the wild-type of strain NCIMB 10586 as overexpression plasmids, through electroporation.

### Bioinformatics and statistics analysis

Similar amino acid sequences were searched by NCBI BLAST and aligned by using ClustalW. The protein instability index and molecular mass were computed using ProtParam software (50). Promoter regions and transcriptional start sites (TSS) were detected with Neural Network Promoter Prediction (51). The Mfold web server (Version 3.6) was used to predict the secondary structures of small regulatory RNAs and mRNAs at 28°C (52). In this study, each experiment was conducted three times with triplicate parallels included. All statistical figures were drawn by using Prism software (GraphPad Software, La Jolla, CA, USA). Data are shown as the mean with the standard deviation of three repeats. Statistical differences among groups were detected by one-way ANOVA (*P* < 0.05), followed by Tukey test. Results were considered significant when the *P* value was < 0.05.

### Quantification of mupirocin production

Samples collected from fermentation broth at spaced time points were centrifuged at 13,000 rpm for 10 minutes. The supernatant was filtered (0.22 µm) for high-performance liquid chromatography (model 1260, Agilent, Santa Clara, USA) detection and stored at −20°C when not in use. The process was conducted by methods described previously (8)

### Signal assay and relative quantification of *N*-acyl-homoserine lactone

It was confirmed that the majority of AHLs in *Pseudomonas* sp. NCIMB 10586 belongs to long-chain homoserine lactone (HSL), and 3-oxo-C_10_-AHL is synthesized in the presence of MupI (7). Here, a signal assay for long-chain AHL was carried out to determine the influences of the relative genes on quorum sensing effects. *A. tumefaciens* NTL4 (pZLR4) and 3-oxo-C_10_-HSL were applied as indicator strain and positive control, respectively. For relative quantification of the main signal, the AHL extraction, sample preparation, and high-performance liquid chromatography-electrospray ionization mass spectrometry (HPLC-ESIMS) analyses were conducted as described (8). 3-oxo-C_10_-AHL was used as the standard molecule. All samples were diluted at the same factor.

### Construction of the xyLE fusion reporter plasmids and assays of XylE activity

To assess the influences of the *gacA* mutant and *rsmY/Z* double mutation on *mupZ*, *mupR,* and *mupI* expressions, the promoter region and 5’UTR starting from predicted TSS, together with promising conserved motifs, of *mupZ*, *mupR*, and *mupI*, were fused in front of a *xylE* gene of the reporter plasmid to generate the transcriptional and translational dual reporters and translational reporters. The *mupZ’-’xylE* transcriptional and translational dual reporter (pSEVA-*mupZ*) was constructed by cloning the 365-bp fragment upstream of the translational start codon into the pSEVA plasmid at the *Eco*R I/*Hind* III sites. Similarly, the fragment covering the promoter region and operator was inserted into pSEVA to generate dual reporters pSEVA-*mupA* (−399 bp relative to translational start codon, the same below), pSEVA-*mupR* (−191 bp), and pSEVA-*mupI* (−132 bp), respectively. For translational reporters, the *mupRo-xylE* (−110 to +30) and *mupIo-xylE* (−50 to +81) reporters were constructed.

The NCIMB 10586 wild-type and its mutants carrying different reporter plasmids were cultivated in MP medium at the same condition. XylE enzymatic assay was conducted as described by Wang et al*.* (53) with some changes for cell lysis. Briefly, cell samples (1 mL each) were collected at 9-hour, 12-hour, and 15-hour time points and resuspended with 850 µL cold sample buffer (100 mM phosphate buffer pH 7.5, 0.5 M EDTA, pH 8.0, 10% (vol/vol) acetone), 50 µL 1% (wt/wt) SDS, and 100 µL chloroform, followed by sonication in an ultrasonic cleaner containing ice water for 30 minutes. The samples were then centrifuged, and 4 µL supernatant was added to 96-well plates containing 200 µL assay buffer. The plate was incubated at 30°C, and absorbances of A_375_ every 2-minute interval from 0 to 40 minutes were recorded by a microplate reader (TECAN Spark). Protein content was measured by Bradford reagent assay (54). The specific activity (mU/mg) was calculated as described by Kieser et al. (55).

### Quantitative real-time PCR (qRT-PCR) analysis

The relative expression level of related genes was assessed by qRT-PCR between the mutants and the wild-type cultured under the same condition as the methods used for mupirocin quantification. Primers are described in Table S2. Culture samples at the midlogarithmic growth phase (12 hours) were collected for RNA extraction and qRT-PCR process as described (56). Each biological sample was analyzed three times. The 16S rRNA gene was used as a reference. The relative transcript level of selected genes was calculated by the 2^−*ΔΔCT*^ method (57).

### Purification of RsmA, RsmE, RsmI, RsmF, and RsmN proteins

Entire coding regions of *rsmA*, *rsmE*, *rsmI*, and *rsmF* genes without the termination codon were amplified and integrated into the *Nco*I/*Xho*I *loci* of pET28a to generate the expression plasmids pET-*rsmA*, pET-*rsmE*, pET-*rsmI*, pET-*rsmF,* and pET-*rsmN*, respectively. These plasmids, in which each protein carries a six-histidine label at the C-terminal, were introduced into *E. coli* BL21 (DE3). The subsequent procedures including cell cultivation, induction, and cell harvesting were conducted as previously described (58). The harvest cells were resuspended in binding buffer (25 mM Tris, 300 mM NaCl, 20 mM imidazole, 1 mM PMSF, 100 µM DTT, and 1% Triton X-100) and lysed by sonication under low temperature. The Rsm-His6 proteins were separated from the lysed supernatant by an Ni-NTA agarose column (Sangon Biotech) and further purified by gel filtration chromatography (GE healthcare) according to the standard protocol.

### Synthesis of RNA probes

The *mupRo* RNA used in EMSA was produced by an ABI 394 synthesizer (Applied Biosystems) and/or biotinylated in 3’-end by TaKaRa (Dalian, China). Purification of RNA products was carried out by nondenaturing anion-exchange HPLC.

### EMSA of RNA–protein interaction

EMSA was performed with the RNA EMSA kit (Thermo, No. 20158) as previously reported (55). The biotinylated RNAs (10 nM) were mixed with different concentration gradients (0–1 μM) of RsmE, RsmI, RsmF, and RsmN and then incubated at 28°C for 30 minutes. Electrophoresis separation and the biotinylated band gel transformation were conducted as described by Wang et al. (30). Subsequently, RNA and RNA-Rsm protein bands on the nylon membrane (Thermo, No. 77016) were visualized by using the Thermo Detection Module (No. 89880). Finally, the bands on membrane were exposed to X-ray film.

## SUPPLEMENTAL MATERIAL

Supplemental material aem.01896-24-s0001.docx Figures S1 to S6; Tables S1 and S2.

ASM does not own the copyrights to Supplemental Material that may be linked to, or accessed through, an article. The authors have granted ASM a non-exclusive, world-wide license to publish the Supplemental Material files. Please contact the corresponding author directly for reuse.
