# Supplementary material for: Global Gac/Rsm regulatory system activates the biosynthesis of mupirocin by controlling the MupR/I quorum sensing system in Pseudomonas sp. NCIMB 10586
Source: Appl Environ Microbiol. 2025 Jan 23;91(2):e01896-24. doi: 10.1128/aem.01896-24 (PMC11837529; doi:10.1128/aem.01896-24)
Supplement: Supplemental material — Figures S1 to S6; Tables S1 and S2. [file aem.01896-24-s0001.docx]

**Supplementary material**

**Global Gac/Rsm regulatory systems activate the biosynthesis of mupirocin by controlling the MupR/I quorum sensing system in *Pseudomonas* sp. NCIMB 10586**

Yuyuan Cai ^a^, Peng Huang ^a^, Vittorio Venturi ^c,d^, Runyao Xiong ^a^, Zheng Wang ^a^, Wei Wang ^a^, Xianqing Huang ^a,^*, Hongbo Hu ^a^, Xuehong Zhang ^a,b,^*

**Author affiliations**

^a^ State Key Laboratory of Microbial Metabolism, School of Life Sciences and Biotechnology, Shanghai Jiao Tong University, Shanghai, 200240, China

^b^ National Experimental Teaching Center for Life Sciences and Biotechnology, Shanghai Jiao Tong University, Shanghai, 200240, China

^c^ International Centre for Genetic Engineering and Biotechnology, Area Science Park, Padriciano 99, Trieste, 34149, Italy

^d^ African Genome Center, University Mohammed VI Polytechnic, Ben Guerir, Morocco

***Corresponding author:** Xuehong Zhang, Xianqing Huang

**E-mail:** xuehzhang@sjtu.edu.cn, xqhuang66@sjtu.edu.cn

**Tel:** +86-21-34204854


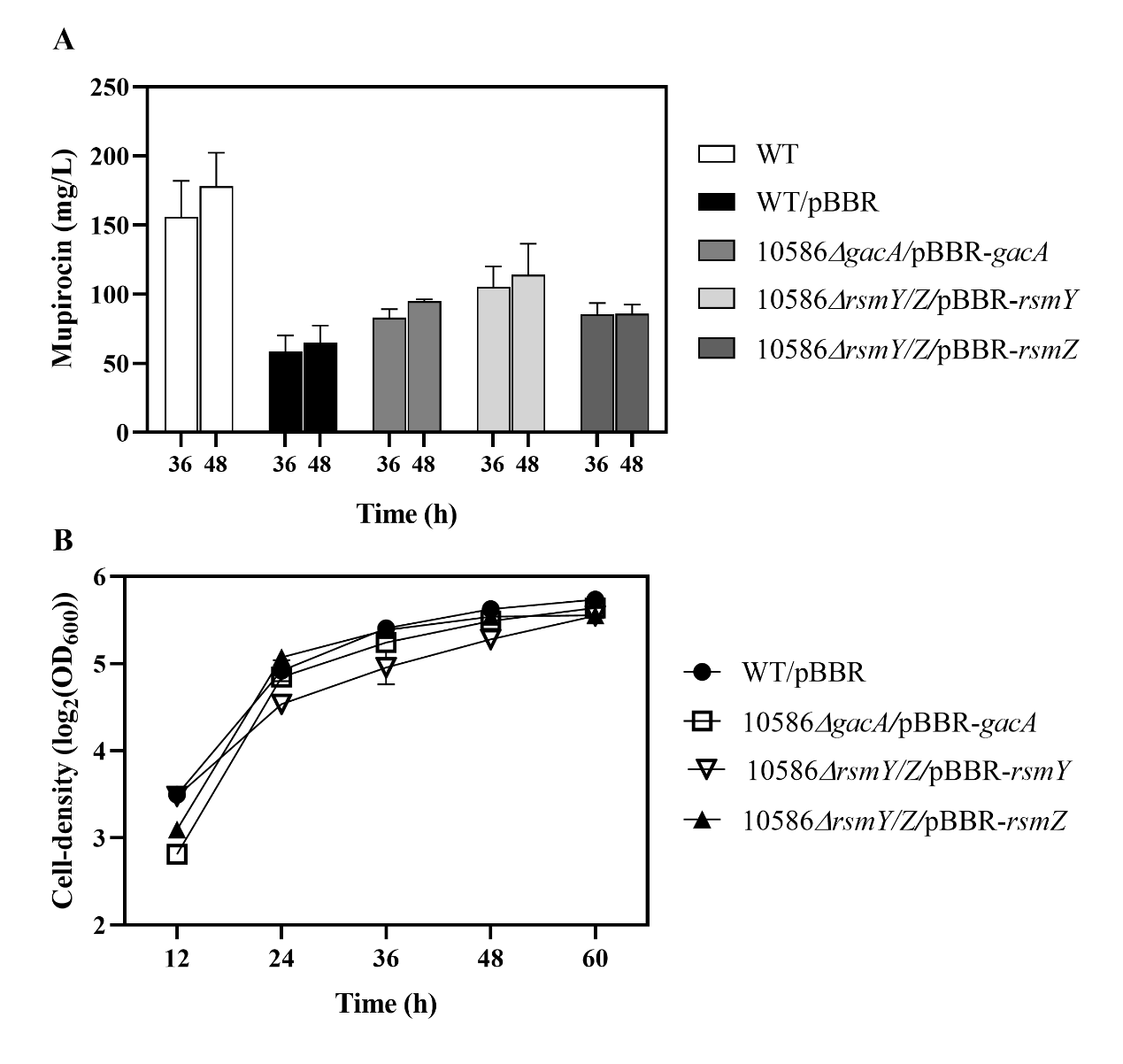


Fig. S1. Mupirocin production **(A)** and cell growth **(B)** of the mutants and the wild-type of strain NCIMB 10586 carrying the expression plasmids or empty pBBR in the gene complementation experiments. The complementation of *gacA* mutant was conducted by introducing the *gacA* expression plasmid (pBBR-*gacA*) into *gacA* mutant. The complementation of *rsmY/Z* double mutant was conducted by introducing the *rsmY* or *rsmZ* expression plasmid (pBBR-*rsmY* or pBBR-*rsmZ*) into 10586*△rsmY/Z*. The wild-type carrying the empty pBBR was used as the control. The production of mupirocin in strain NCIMB 10586 carrying the empty pBBR decreased compared to that of the wild-type strain of plasmid-free.


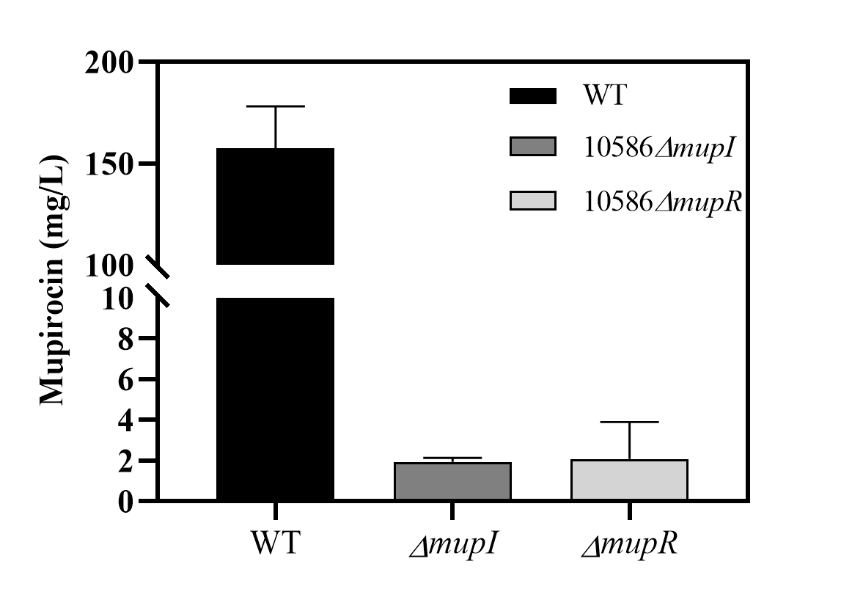


Fig. S2. The mupirocin production of the mutants, 10586*△mupR* and 10586*△mupI*.


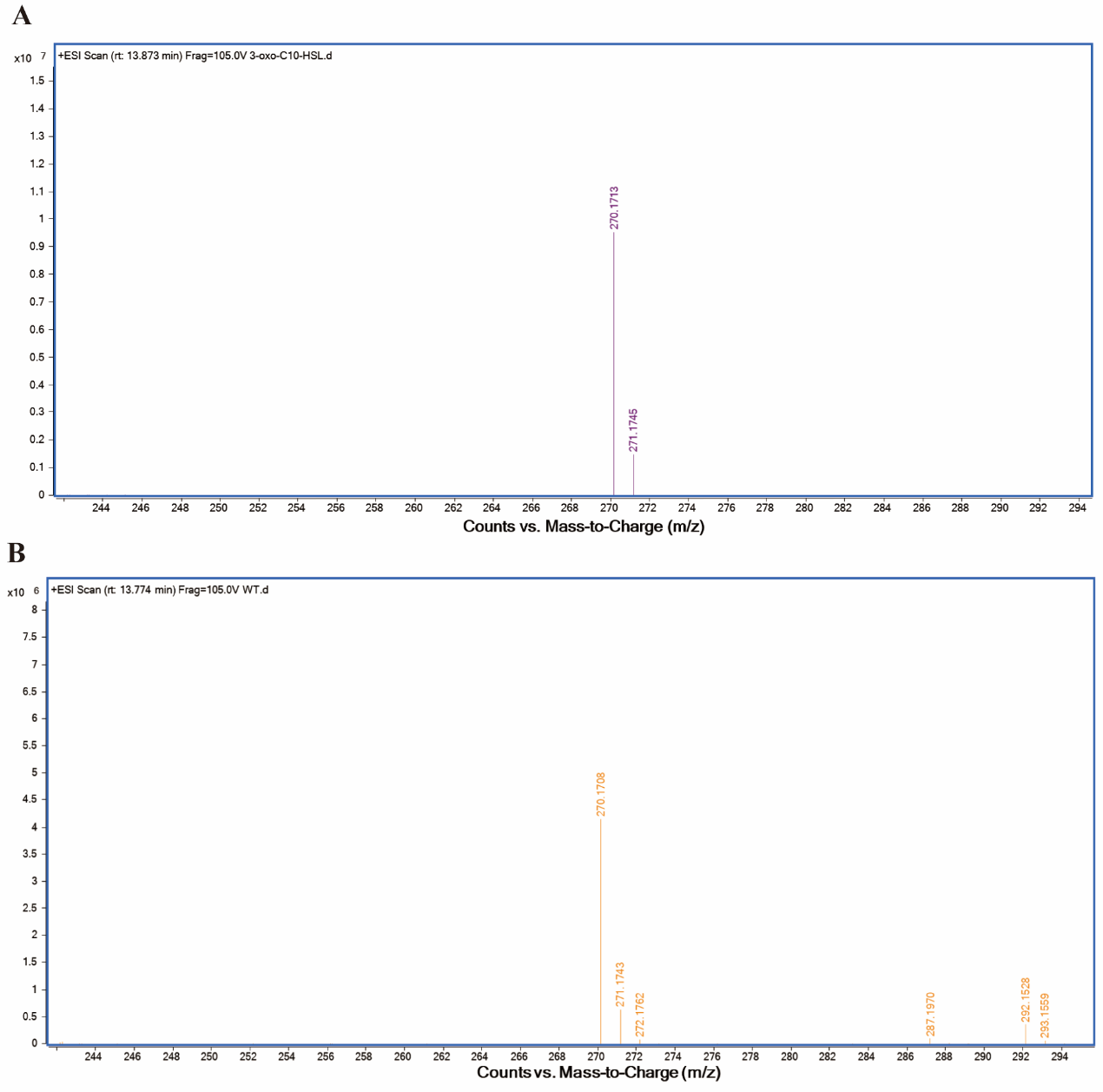


Fig. S3. Positive electrospray HPLC-ESIMS analysis of a standard of 3-oxo-C_10_-HSL (**A**) and the solvent extracts from the wild-type (**B**). The HPLC-ESIMS analysis revealed a peak at m/z 270.17 corresponding to 3-oxo-C_10_-HSL. The abscissa represents the counts vs acquisition time (retention time) and the ordinate the UV absorption abundance. ESI, electron spray ionization.


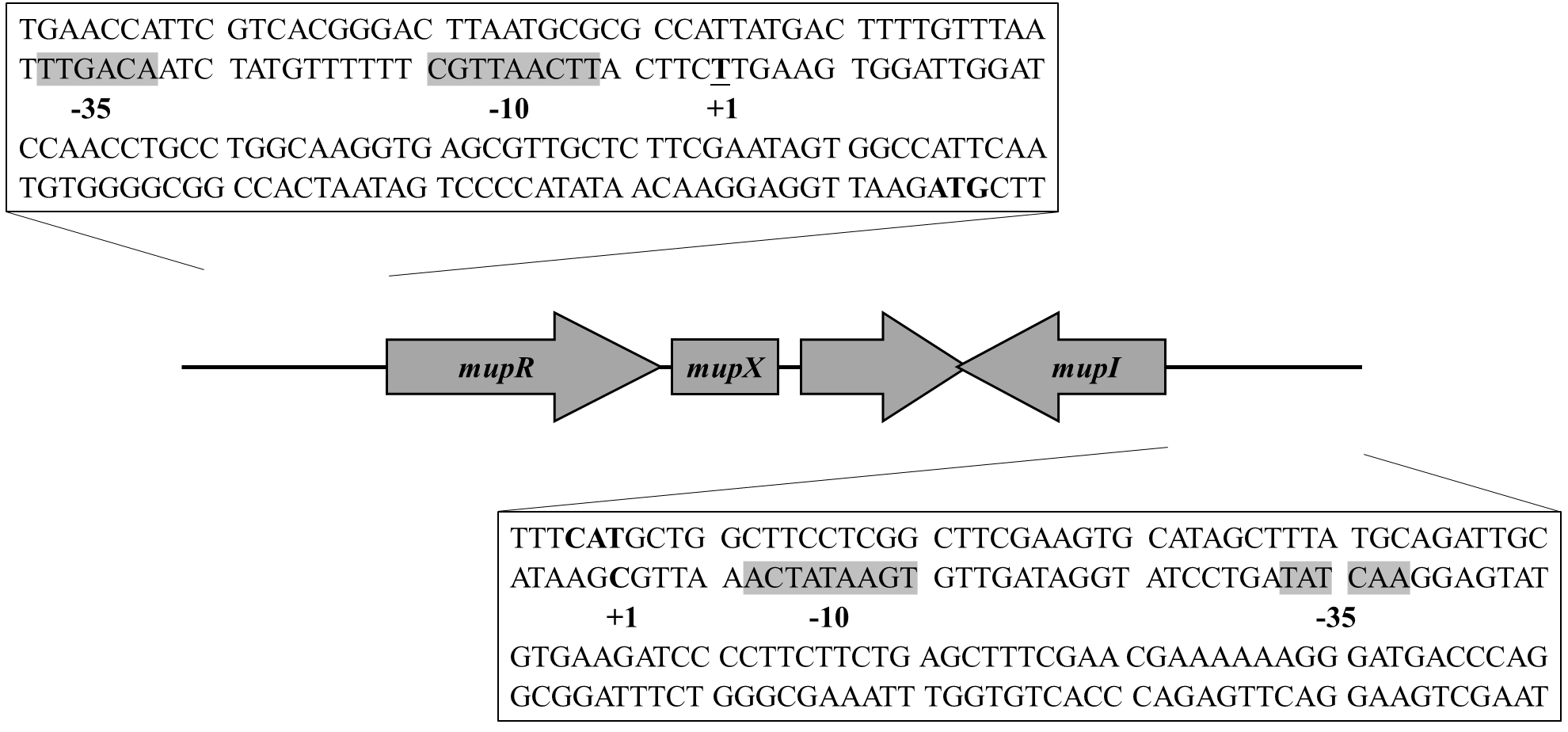


Fig. S4. Prediction of the transcriptional start sites (TSSs) and promoters in the 5’UTR of *mupR* and *mupI*. “+1” refers to the transcription site by prediction.


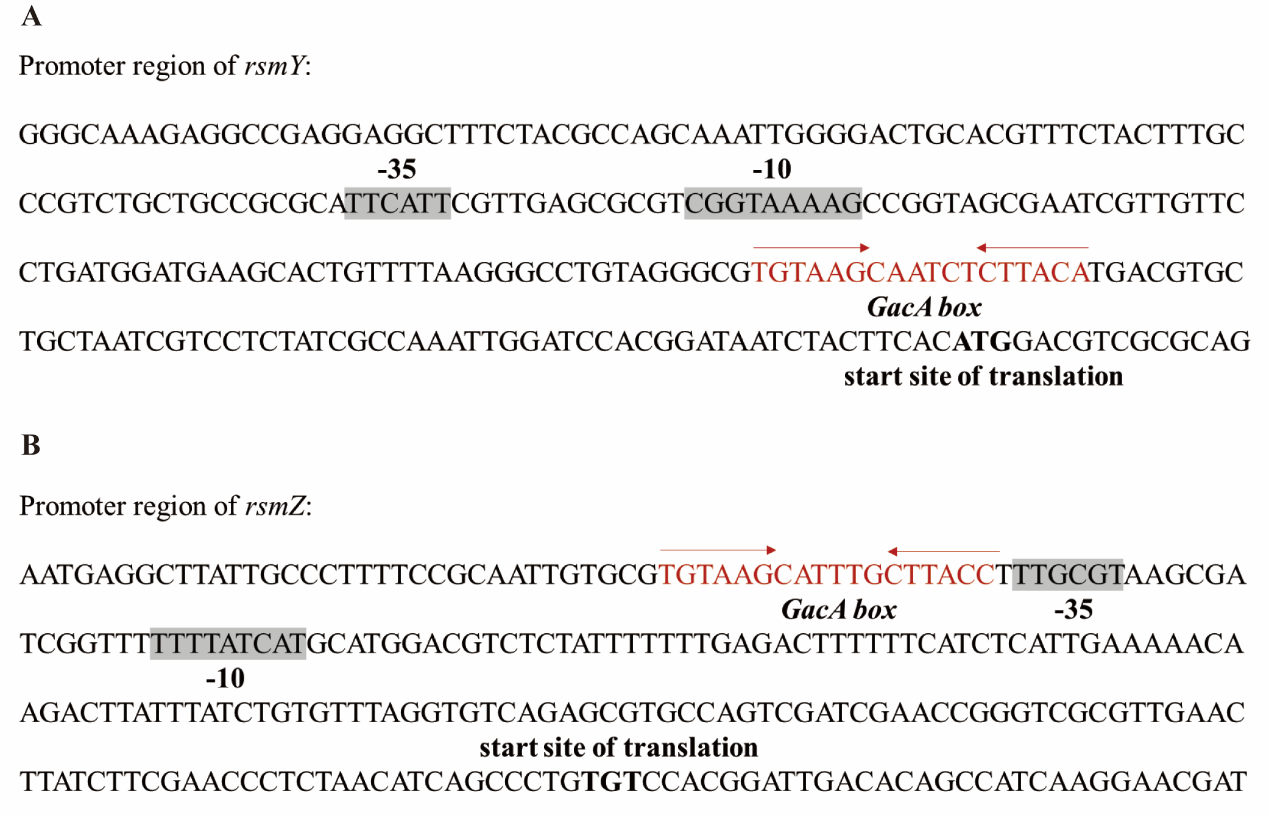


Fig. S5. The promoter regions of *rsm* small regulatory RNAs, (A) *rsmY* and (B) *rsmZ*, in NCIMB 10586.


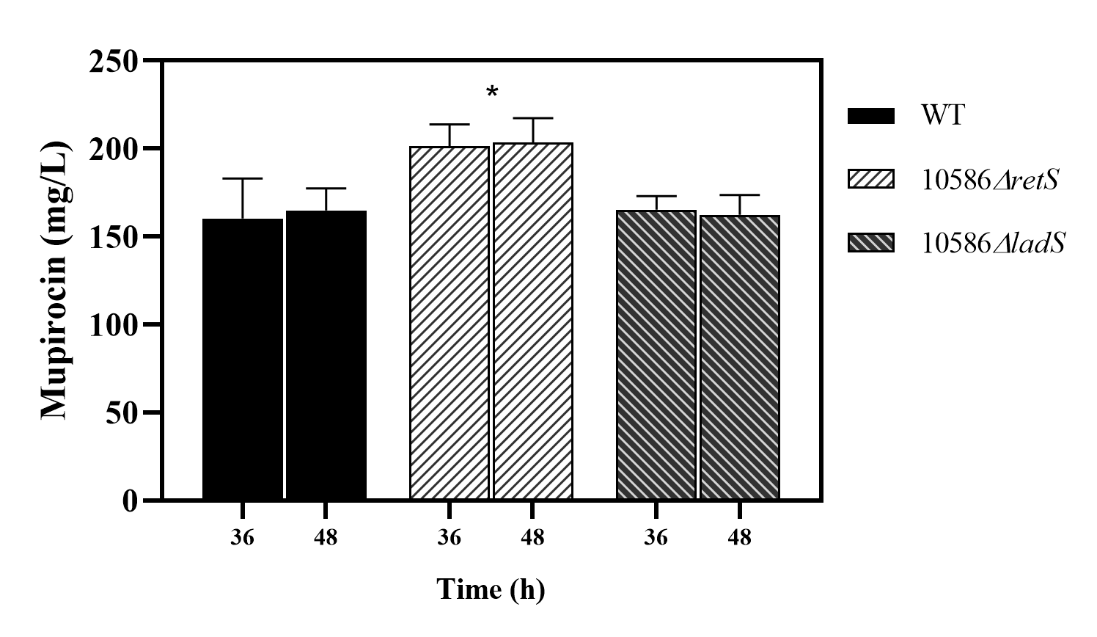


Fig. S6. The mupirocin production of RetS and LadS mutants, respectively.

Table S1 Strains and plasmids used in this study

| **Strains/Plasmids** | **Genotype** | **Reference** |
| --- | --- | --- |
| ***E. coli*** |  |  |
| DH5α | *supE*44 Δ*lac*U169(Φ80 *lacZ*ΔM15) *hsdR17* *recA1* *endA1 gyrA96* *thi-1* *relA1* | (1) |
| S17-1 (*λ*pir) | res^-^ pro mod^+^ integrated copy of RP4, mob^+^ | (2) |
| BL21(DE3) | E. coli B, F-, ompT, hsdSB(rB^-^mB^-^), gal, dcm (DE3) | (1) |
| ***Pseudomonas* sp. strain NCIMB 10586** |  |  |
| NCIMB 10586 | Wild type, Amp^r^ | (3) |
| 10586*△gacA* | In frame deletion of gac*A* | This study |
| 10586*△gacS* | *gacS* mutant, inactivation of *gacS* | This study |
| 10586*△rsmY* | In frame deletion of *rsmY* | This study |
| 10586*△rsmZ* | In frame deletion of *rsmZ* | This study |
| 10586*△rsmY/Z* | In frame deletion of *rsmY* and *rsmZ* | This study |
| 10586*△rsmA* | In frame deletion of *rsmA* | This study |
| 10586*△rsmE* | In frame deletion of *rsmE* | This study |
| 10586*△rsmI* | In frame deletion of *rsmI* | This study |
| 10586*△rsmF* | In frame deletion of *rsmF* | This study |
| 10586*△rsmN* | In frame deletion of *rsmN* | This study |
| 10586*△rsmAE* | Double deletion of *rsmA* and *rsmE* | This study |
| 10586*△rsmAI* | Double deletion of *rsmA* and *rsmI* | This study |
| 10586*△rsmAF* | Double deletion of *rsmA* and *rsmF* | This study |
| 10586*△rsmAN* | Double deletion of *rsmA* and *rsmN* | This study |
| 10586*△rsmEI* | Double deletion of *rsmE* and *rsmI* | This study |
| 10586*△rsmEF* | Double deletion of *rsmE* and *rsmF* | This study |
| 10586*△rsmEN* | Double deletion of *rsmE* and *rsmN* | This study |
| 10586*△rsmIF* | Double deletion of *rsmI* and *rsmF* | This study |
| 10586*△rsmIN* | Double deletion of *rsmI* and *rsmN* | This study |
| 10586*△rsmFN* | Double deletion of *rsmF* and *rsmN* | This study |
| 10586*△rsmAEF* | Triple deletion of *rsmA, rsmE,* and *rsmF* | This study |
| 10586*△rsmAEI* | Triple deletion of *rsmA, rsmE,* and *rsmI* | This study |
| 10586*△rsmAIF* | Triple deletion of *rsmA, rsmI,* and *rsmF* | This study |
| 10586*△rsmAEN* | Triple deletion of *rsmA, rsmE,* and *rsmN* | This study |
| 10586*△rsmEIF* | Triple deletion of *rsmE, rsmI,* and *rsmF* | This study |
| 10586*△rsmAIN* | Triple deletion of *rsmA, rsmI,* and *rsmN* | This study |
| 10586*△rsmAFN* | Triple deletion of *rsmA, rsmF,* and *rsmN* | This study |
| 10586*△rsmEFN* | Triple deletion of *rsmE, rsmF,* and *rsmN* | This study |
| 10586*△rsmEIN* | Triple deletion of *rsmA, rsmE,* and *rsmI* | This study |
| 10586*△rsmIFN* | Triple deletion of *rsmI, rsmE,* and *rsmN* | This study |
| 10586*△rsmAEIF* | Quadruple deletion of *rsmA, rsmE,* *rsmI,* and *rsmF* | This study |
| 10586*△rsmAEFN* | Quadruple deletion of *rsmA, rsmE, rsmF,* and *rsmN* | This study |
| 10586*△rsmAEIN* | Quadruple deletion of *rsmA, rsmE, rsmI,* and *rsmN* | This study |
| 10586*△rsmAIFN* | Quadruple deletion of *rsmA, rsmI, rsmF,* and *rsmN* | This study |
| 10586*△rsmEIFN* | Quadruple deletion of *rsmE, rsmI,* *rsmF,* and *rsmN* | This study |
| 10586*△rsmAEIFN* | Quintuple deletion of *rsmA, rsmE,* *rsmI, rsmF,* and *rsmN* | This study |
| **Plasmids** |  |  |
| pk18mob*sacB* | Broad-host-range gene replacement vector; *sacB*, Km^r^ | Lab stock |
| pK18-*gacA* | pK18mob*sacB* with *Eco*R I-*Hind* Ⅲ insert of 500 bp and 500 bp segments flanking *gacA*, Km^r^ | This study |
| pK18-*gacS* | pK18mob*sacB* with *Eco*R I-*Hind* Ⅲ insert of 500 bp and 500 bp segments flanking *gacS*, Km^r^ | This study |
| pK18-*rsmY* | pK18mob*sacB* with *Eco*R I-*Hind* Ⅲ insert of 514 bp and 500 bp segments flanking *rsmY*, Km^r^ | This study |
| pK18-*rsmZ* | pK18mob*sacB* with *Eco*R I-*Hind* Ⅲ insert of 531 bp and 504 bp segments flanking *rsmZ*, Km^r^ | This study |
| pK18-*rsmA* | pK18mob*sacB* with *Eco*R I-*Hind* Ⅲ insert of 500 bp and 507 bp segments flanking *rsmA*, Km^r^ | This study |
| pK18-*rsmE* | pK18mob*sacB* with *Eco*R I-*Hind* Ⅲ insert of 497 bp and 527 bp segments flanking *rsmE*, Km^r^ | This study |
| pK18-*rsmI* | pK18mob*sacB* with *Eco*R I-*Hind* Ⅲ insert of 500 bp and 509 bp segments flanking *rsmI*, Km^r^ | This study |
| pK18-*rsmF* | pK18mob*sacB* with *Eco*R I-*Hind* Ⅲ insert of 538 bp and 473 bp segments flanking *rsmF*, Km^r^ | This study |
| pK18-*rsmN* | pK18mob*sacB* with *Eco*R I-*Hind* Ⅲ insert of 472 bp and 519 bp segments flanking *rsmN*, Km^r^ | This study |
| pBBR1MCS-2 | Overexpression vector, broad host range, oriV(pBBR1), P*_lac_*, Km^r^ | Lab stock |
| pBBR-*gacA* | pBBR1MCS-2 carrying the entire 642 bp *gacA* ORF | This study |
| pBBR-*rsmY* | pBBR1MCS-2 carrying the entire 118 bp *rsmY* sRNA | This study |
| pBBR-*rsmZ* | pBBR1MCS-2 carrying the entire 132 bp *rsmZ* sRNA | This study |
| pBBR-*rsmA* | pBBR1MCS-2 carrying the entire 189 bp *rsmA* ORF | This study |
| pBBR-*rsmE* | pBBR1MCS-2 carrying the entire 195 bp *rsmE* ORF | This study |
| pBBR-*rsmI* | pBBR1MCS-2 carrying the entire 204 bp *rsmI* ORF | This study |
| pBBR-*rsmF* | pBBR1MCS-2 carrying the entire 192 bp *rsmF* ORF | This study |
| pBBR-*rsmN* | pBBR1MCS-2 carrying the entire 231 bp *rsmN* ORF | This study |
| pSEVA644 | Cloning vector, oriV (pRO1600), Gm^r^ | (4) |
| pSEVA-*mupZ’-’xylE* | pSEVA644 containing *mupZ-xylE* transcriptional and translational fusion, Gm^r^ | This study |
| pSEVA-*mupA’-’xylE* | pSEVA644 containing *mupA-xylE* transcriptional and translational fusion, Gm^r^ | This study |
| pSEVA-*mupR’-’xylE* | pSEVA644 containing *mupR-xylE* transcriptional and translational fusion, Gm^r^ | This study |
| pSEVA-*mupI’-’xylE* | pSEVA644 containing *mupI-xylE* transcriptional and translational fusion, Gm^r^ | This study |
| pSEVT | A derivative plasmid of pSEVA644, P*_tac_*, oriV (pRO1600), Gm^r^ | This study |
| pSEVT-*mupRo-xylE* | pSEVT containing *mupR-xylE* translational fusion, Gm^r^ | This study |
| pSEVT-*mupIo-xylE* | pSEVT containing *mupI-xylE* translational fusion, Gm^r^ | This study |
| pET28a | T7 expression vector, Km^r^ | Novagen |
| pET-*rsmA* | Intact *rsmA* CDS of *P. fluorescens* NCIMB 10586 | This study |
| pET-*rsmE* | Intact *rsmE* CDS of *P. fluorescens* NCIMB 10586 | This study |
| pET-*rsmI* | Intact *rsmI* CDS of *P. fluorescens* NCIMB 10586 | This study |
| pET-*rsmF* | Intact *rsmF* CDS of *P. fluorescens* NCIMB 10586 | This study |
| pET-*rsmN* | Intact *rsmN* CDS of *P. fluorescens* NCIMB 10586 | This study |

Table S2 Primers used in this study

| Purpose | Name | Sequence (5’-3’) |
| --- | --- | --- |
| **For mutant construction** | |  |
| *gacA* | gacA-F1 | TATGACATGATTACGAATTCGAGCGTGTCTGTCGCGTA |
|  | gacA-R1 | GCAGACACCTCGCGATATG |
|  | gacA-F2 | CATATCGCGAGGTGTCTGCCATGACTACGCCGTTTGATCC |
|  | gacA-R2 | CGACGGCCAGTGCCAAGCTTCTGGTATTGCAGGCACGG |
| *gacS* | gacS-F1 | TATGACATGATTACGAATTCATGGGGATAAAAGGCCGC |
|  | gacS-R1 | GCGAACAGGCTGCGGTAG |
|  | gacS-F2 | CTACCGCAGCCTGTTCGCACGCCATGGCCAATGAAA |
|  | gacS-R2 | CGACGGCCAGTGCCAAGCTTTCAGGCGCTGACTCTGGC |
| *rsmY* | rsmY-F1 | TATGACATGATTACGAATTCCCCTCGACATTGGCATCAG |
|  | rsmY-R1 | CTTTTGCCCGCGAAAAAGTCAG |
|  | rsmY-F2 | CTGACTTTTTCGCGGGCAAAAGGTGAAGTAGATTATCCGTGGATCC |
|  | rsmY-R2 | CGACGGCCAGTGCCAAGCTTGATGGGACGCAAGATCGTC |
| *rsmZ* | rsmZ-F1 | TATGACATGATTACGAATTCATGCTGCGGGCTTTTAGC |
|  | rsmZ-R1 | CGCCTGTAGAAAAGTAAAAACGCT |
|  | rsmZ-F2 | AGCGTTTTTACTTTTCTACAGGCGCAGGGCTGATGTTAGAGGG |
|  | rsmZ-R2 | CGACGGCCAGTGCCAAGCTTGTGCACATCTGCTCCAAC |
| *rsmA* | rsmA-F1 | TATGACATGATTACGAATTCCCGATTTCACCTTCACCG |
|  | rsmA-R1 | GCCTTTCTCCTCACGCAT |
|  | rsmA-F2 | ATGCGTGAGGAGAAAGGCGGACGAAGAACCAAGCCT |
|  | rsmA-R2 | CGACGGCCAGTGCCAAGCTTAGGCGGGCTTTTTGTTTG |
| *rsmE* | rsmE-F1 | TATGACATGATTACGAATTCTCATCCTTGCCGTCGACCTTC |
|  | rsmE-R1 | GGACTTCTCCTTGATTACTTTTAAGGAC |
|  | rsmE-F2 | GTCCTTAAAAGTAATCAAGGAGAAGTCCAACGCCTCCAGTAGCCAG |
|  | rsmE-R2 | CGACGGCCAGTGCCAAGCTTGTGTGGTTTTCTGATGGGCAC |
| *rsmI* | rsmI-F1 | TATGACATGATTACGAATTCAGCAACTACAACGTGGCCAAG |
|  | rsmI-R1 | GGCGTGTGTCCTTTTCAAGTAGC |
|  | rsmI-F2 | GCTACTTGAAAAGGACACACGCCGCGGGGTTTTCAGTCGAACAG |
|  | rsmI-R2 | CGACGGCCAGTGCCAAGCTTGTACCCATATCGACAGCCTGGC |
| *rsmF* | rsmF-F1 | TATGACATGATTACGAATTCGACATGCGGATTTTGATAGTGGCC |
|  | rsmF-R1 | GTAAGGTGCCCATGAGCATAATCG |
|  | rsmF-F2 | CGATTATGCTCATGGGCACCTTACTGGGTATCCTTTCTGGTTAGCTCC |
|  | rsmF-R2 | CGACGGCCAGTGCCAAGCTTCGGCAAAACACATCACCACGA |
| *rsmN* | rsmN-F1 | TATGACATGATTACGAATTCGATCTCTGTCAGGACCACGCC |
|  | rsmN-R1 | CAGACCGTCCCGCGATTGATAAG |
|  | rsmN-F2 | CTTATCAATCGCGGGACGGTCTGCTTGATCTGAATTTCGTCGTCGATACG |
|  | rsmN-R2 | CGACGGCCAGTGCCAAGCTTACTGCAGCTTCAGACGAGTGTG |
| **For complementation and overexpression** | | |
| linearized pBBR1MCS-2 | pBBR-F | agctgtttcctgtgtgaaattgttatc |
|  | pBBR-R | aatggcgaatggaaattgtaagcg |
| pBBR-*gacA* | gacAc-F | gataacaatttcacacaggaaacagctTTGATTAGGGTGCTAGTAGTCGATGATCATG |
|  | gacAc-R | cgcttacaatttccattcgccattTCAGGCGCTGGCGTCCAC |
| pBBR-*rsmY* | rsmYc-F | gataacaatttcacacaggaaacagctATGGACGTCGCGCAGGAAG |
|  | rsmYc-R | cgcttacaatttccattcgccattAAAACCCCGCCGAAGCGGGG |
| pBBR-*rsmZ* | rsmZc-F | gataacaatttcacacaggaaacagctTGTCCACGGATTGACACAGCC |
|  | rsmZc-R | cgcttacaatttccattcgccattAAAAAAGGGGCGGTATGACCC |
| pBBR-*rsmA* | rsmAc-F | gataacaatttcacacaggaaacagctATGCTGATTCTGACTCGTCGTTG |
|  | rsmAc-R | cgcttacaatttccattcgccattTTAAAGGCTTGGTTCTTCGTCCTTC |
| pBBR-*rsmE* | rsmEc-F | gataacaatttcacacaggaaacagctATGCTTATACTCACCCGCAAAGTTG |
|  | rsmEc-R | cgcttacaatttccattcgccattTCAAGGCGTCTGGTTTTTGTCG |
| pBBR-*rsmI* | rsmIc-F | GataacaatttcacacaggaaacagctATGCTGGTCTTAAGCCGCAC |
|  | rsmIc-R | cgcttacaatttccattcgccattCTAAGGATTGGGTACCGACACG |
| pBBR-*rsmF* | rsmFc-F | gataacaatttcacacaggaaacagctTTGCTAGTTGTCACCAGAACACC |
|  | rsmFc-R | cgcttacaatttccattcgccattCTATTCCAGTGCATCGGAACCTATAG |
| pBBR-*rsmN* | rsmNc-F | gataacaatttcacacaggaaacagctTTGCTACTTATCACTAGGAGGACCG |
|  | rsmNc-R | cgcttacaatttccattcgccattTCATGACCGATTTTTTGGCAATGG |
| **For construction of XylE reporters** | | |
| pSEVA-*mupZ’-’xylE* | mupZ*’*-F1 | TTAATTGACACCATGAATTCgttagaataccggcctgtcacg |
|  | mupZ*’*-R1 | cttcggccgttccgttagaac |
|  | mupZ*’*-F2 | gttctaacggaacggccgaagatgaacaaaggtgtaatgcgaccg |
|  | xylE-R2 | CGACGCGGCCGCAAGCTTtcaggtcagcacggtcatgaatc |
| pSEVA-*mupA’-’xylE* | mupA*’*-F1 | TTAATTGACACCATGAATTCatgaatcgcacctgcatggc |
|  | mupA*’*-R1 | gtcttgaggcctcttgtagatacatc |
|  | mupA*’*-F2 | gatgtatctacaagaggcctcaagacatgaacaaaggtgtaatgcgaccg |
|  | xylE-R2 | CGACGCGGCCGCAAGCTTtcaggtcagcacggtcatgaatc |
| pSEVA-*mupR’-’xylE* | mupR*’*-F1 | TTAATTGACACCATGAATTCaccattcgtcacgggacttaatg |
|  | mupR*’*-R1 | cttaacctccttgttatatggggac |
|  | mupR*’*-F2 | gtccccatataacaaggaggttaagatgaacaaaggtgtaatgcgaccg |
|  | xylE-R2 | CGACGCGGCCGCAAGCTTtcaggtcagcacggtcatgaatc |
| pSEVA-*mupI’-’xylE* | mupI*’*-F1 | TTAATTGACACCATGAATTCaaacgcccaactcccgc |
|  | mupI*’*-R1 | gctggcttcctcggcttcg |
|  | mupI*’*-F2 | cgaagccgaggaagccagcatgaacaaaggtgtaatgcgaccg |
|  | xylE-R2 | CGACGCGGCCGCAAGCTTtcaggtcagcacggtcatgaatc |
| pSEVT-*mupRo-xylE* | mupRo-F | GTGAGCGGATAACAATTTCACttgaagtggattggatccaacctgc |
|  | mupRo-R | cggtcgcattacacctttgttcgtctcacacatcagaatgtcttcaagc |
| pSEVT-*mupIo-xylE* | mupIo-F | GTGAGCGGATAACAATTTCACgcttatgcaatctgcataaagctatgc |
|  | mupIo-R | cggtcgcattacacctttgttgaagattgaagcccgaagtttatgc |
| **For qPCR** |  |  |
| 16S-qPCR | 16S-F | TGAAGTCGGAATCGCTAGTAATCGC |
|  | 16S-R | TTGTTACGACTTCACCCCAGTCATG |
| *rsmY*-qPCR | rsmY-F | TCCCTGACATCCATTTCACTCCG |
|  | rsmY-R | GCAGGAAGCGCAAAGCAACAA |
| *rsmZ*-qPCR | rsmZ-F | CGGTATGACCCGCCCACATTTT |
|  | rsmZ-R | GATTGACACAGCCATCAAGGAACGA |
| *mupZ*-qPCR* | mupZ-F | ATGCCTGTACTACGGTTTCAGCTTC |
|  | mupZ-R | CATAGCGAACAATTGAAGCCAGGTG |
| *mupA*-qPCR | mupA-F | cttcacgatgttgtacggcattga |
|  | mupA-R | cagtgagctgcggatatagtctttg |
| *mupR*-qPCR* | mupR-F | CATATCACCACGTTATCTGGAGGGC |
|  | mupR-R | CAAGGCGTCGTAACCATCTATCTCC |
| *mupX*-qPCR* | mupX-F | TAACCCCTGGAACTTGGAATTGTCG |
|  | mupX-R | CATCACTCCCCGCAATCAGGATATC |
| *mupI*-qPCR* | mupI-F | AGACATTCTGATGTGTGAGACGCTG |
|  | mupI-R | CTCATACAATTGCTGTTCGGGCTTG |

*Originated from Huang et al. (2021)

Reference

1. Sambrook J, Russell DW. 2001. Molecular cloning: a laboratory manual, third ed. Cold Spring Harbor Laboratory Press, New York.

2. Schafer A, Kalinowski J, Simon R, Seep-Feldhaus AH, Puhler A. 1990. High-frequency conjugal plasmid transfer from gram-negative *Escherichia coli* to various gram-positive coryneform bacteria. J Bacteriol 172:1663-1666. https://doi.org/10.1128/jb.172.3.1663-1666.1990.

3. Whatling CA, Hodgson JE, Burnham MKR, Clarke NJ, Franklin FCH, Thomas CM. 1995. Identification of a 60 kb region of the chromosome of *Pseudomonas fluorescens* NCIB 10586 required for the biosynthesis of pseudomonic acid (mupirocin). Microbiology 141:973-982. https://doi.org/10.1099/13500872-141-4-973.

4. Silva-Rocha, R., Martínez-García, E., Calles, B., Chavarría, M., Arce-Rodríguez, A., de las Heras, A., Páez-Espino, A.D., Durante-Rodríguez, G., Kim, J., Nikel, P.I., Platero, R., de Lorenzo V., 2013. The Standard European Vector Architecture (SEVA): a coherent platform for the analysis and deployment of complex prokaryotic phenotypes. Nucleic. Acids Res. 41, 666-675. https://doi.org/10.1093/nar/gks1119.
